# In a zebrafish biomedical model of human Allan-Herndon-Dudley syndrome impaired MTH signaling leads to decreased neural cell diversity

**DOI:** 10.3389/fendo.2023.1157685

**Published:** 2023-05-04

**Authors:** Nádia Silva, Marco António Campinho

**Affiliations:** ^1^ Centre for Marine Sciences of the University of the Algarve, Faro, Portugal; ^2^ Algarve Biomedical Center-Research Institute, University of the Algarve, Faro, Portugal; ^3^ Faculty of Medicine and Biomedical Sciences, University of the Algarve, Faro, Portugal

**Keywords:** maternal thyroid hormone, monocarboxylic acid transporter 8, neurodevelopment, spinal cord, zebrafish, Allan-Herndon-Dudley syndrome (AHDS)

## Abstract

**Background:**

Maternally derived thyroid hormone (T3) is a fundamental factor for vertebrate neurodevelopment. In humans, mutations on the thyroid hormones (TH) exclusive transporter monocarboxylic acid transporter 8 (*MCT8*) lead to the Allan-Herndon-Dudley syndrome (AHDS). Patients with AHDS present severe underdevelopment of the central nervous system, with profound cognitive and locomotor consequences. Functional impairment of zebrafish T3 exclusive membrane transporter Mct8 phenocopies many symptoms observed in patients with AHDS, thus providing an outstanding animal model to study this human condition. In addition, it was previously shown in the zebrafish *mct8* KD model that maternal T3 (MTH) acts as an integrator of different key developmental pathways during zebrafish development.

**Methods:**

Using a zebrafish Mct8 knockdown model, with consequent inhibition of maternal thyroid hormones (MTH) uptake to the target cells, we analyzed genes modulated by MTH by qPCR in a temporal series from the start of segmentation through hatching. Survival (TUNEL) and proliferation (PH3) of neural progenitor cells (*dla*, *her2*) were determined, and the cellular distribution of neural MTH-target genes in the spinal cord during development was characterized. In addition*, in-vivo* live imaging was performed to access NOTCH overexpression action on cell division in this AHDS model. We determined the developmental time window when MTH is required for appropriate CNS development in the zebrafish; MTH is not involved in neuroectoderm specification but is fundamental in the early stages of neurogenesis by promoting the maintenance of specific neural progenitor populations. MTH signaling is required for developing different neural cell types and maintaining spinal cord cytoarchitecture, and modulation of NOTCH signaling in a non-autonomous cell manner is involved in this process.

**Discussion:**

The findings show that MTH allows the enrichment of neural progenitor pools, regulating the cell diversity output observed by the end of embryogenesis and that Mct8 impairment restricts CNS development. This work contributes to the understanding of the cellular mechanisms underlying human AHDS.

## 1 Introduction

During the early stages of vertebrate development, the embryonic naïve system cannot endogenously produce thyroid hormones (TH), thus depending on a precise supply of maternal-derived TH, which is essential for proper central nervous system (CNS) development. Maternally produced prohormone thyroxine (T4) must be converted locally into the active form triiodothyronine (T3) by deiodinase 2. T3 acts in target cells by binding to thyroid hormone receptors (TRs), which regulates target gene expression (1). TH importantly influences neurodevelopment during the fetal period and regulates processes involved in the formation of the cytoarchitecture of the brain, such as proliferation, migration and myelination and neuronal and glial cell differentiation (2–6). Maternal TH (MTH) deprivation outcomes in offspring are various and mainly depend on the timing and severity of the deficiency (3, 7).

The genetic responses to T3 in specific cellular contexts have been identified (8, 9), and several phenotypic outcomes arising from inappropriate levels of TH supply were found (7, 10). However, the underlying cellular and developmental mechanisms of action are less understood. Furthermore, a key feature of TH developmental action is the strict windows of time when the hormone acts, which determine the biological outcome (11, 12).

The importance of MTH transport by monocarboxylate transporter 8 (MCT8, SLC16A2), in human neurodevelopment is highlighted by the severe global neurological impairment observed in subjects with the rare human disease Allan-Herndon-Dudley-Syndrome (AHDS) (13, 14). This disease is characterized by developmental delay, reduced myelination, intellectual disability, poor language and walking skills, hypotonia, and a reduced life span (15), and histopathological outcomes can be identified from fetal stages (16). The severeness of the phenotypic outcome varies among patients (17–20), and could be related to the residual functionality of the mutant MCT8 protein and, consequently, the efficiency of TH transport into the target cells (21).

In the fetal and adult human brains, MCT8 is expressed in the blood-brain barrier (BBB) and blood-cerebrospinal fluid barrier (BCSFB). In fetal stages, MCT8 localizes in ependymal cells, tanycytes, neurons, and cells of the ventricular (VZ) and subventricular (SVZ) zones, the proliferative areas of the brain (22, 23).

The consequences of AHDS highlight the fundamental role of MTH on vertebrate neurodevelopment. Until recently, postnatal treatment of these patients with TH supplementation, with the TH analogs, TRIAC (24) or DITPA that do not require transport by MCT8 results in better thyroid function tests, improving hypermetabolism. However, no motor or cognitive skills improvement was observed (25). Very recently, prenatal treatment using levothyroxine (LT4) ameliorated the neuromotor and neurocognitive function of an AHDS patient (26)

Zebrafish is an established model for AHDS study (27–30). High concentrations of maternally deposited TH have been found in fish eggs (31). Also present in unfertilized eggs are many transcripts of components of the thyroid axis (32). Virtually all known components of the thyroid axis have been characterized in zebrafish, and these are structurally and functionally comparable with higher vertebrates (33, 34). The high degree of conservation between zebrafish *mct8* and its mammalian orthologs (35), points to a conservation of function, albeit zebrafish Mct8 specifically transports T3 at physiological temperature (26°C) and T3 and T4 at human physiological temperature (37°C) (36). Expression of *mct8* in zebrafish is detected from 3hpf with expression increasing through larval stages peaking at 48-96hpf (36, 37). Another advantage of zebrafish to model AHDS is that until 60hpf, there is no endogenous production of TH (38). In the zebrafish model, the developmental action of MTH through Mct8 can be examined without major compensatory mechanisms such as maternal TH compensation, endogenous TH production, or increased uptake by co-expressed TH transporters, as occurs in the mouse model (39), where a similar model of AHDS was only achieved after double KO of *Mct8* and *Oatp1c1* (40–42) or *Mct8* and *D2* (43). More recently, it has been reported the generation of a new mouse model with a human AHDS patient-derived *MCT8* mutation that presents brain hypothyroidism alongside neuro-architectural changes (44). This new mouse model presents similarities to already available zebrafish AHDS models where suppressing Mct8 function (27–29) makes it possible to reproduce many pre-natal neurological consequences observed in human patients with AHDS (16). Previous evidence from zebrafish Mct8 loss of function studies showed that several neural progenitors and neurons depend on MTH for development (27, 45). The spinal cord appears significantly reliant on MTH action for its normal development since, in the absence of functional Mct8, dorsal and medial neurons are mostly lost or show an abnormal morphology and positioning (27). In contrast, ventral spinal cord neurons are favored and increase their number (27). Transcriptomic analysis of zebrafish *mct8* morphant embryos revealed that MTH modulates several critical developmental networks, like Notch, Wnt, and Hh signaling, thus working as an integrative signal (45). Nonetheless, fundamental questions on the action of MTH in zebrafish embryonic development and AHDS remain unanswered. In the present work, we focused on zebrafish spinal cord development and aimed to elucidate three fundamental questions: 1) the developmental time window where MTH action occurs, 2) the types of neural cell populations dependent on MTH signaling, 3) the cellular mechanisms of MTH action.

## 2 Materials and methods

### 2.1 Zebrafish husbandry and spawning

Adult wild-type (AB strain) zebrafish were maintained in standard conditions in the CCMAR fish facility at the University of Algarve (Portugal). Adult fish were kept at a 14 h/10 h light/dark cycle and 28°C. Breeding stock feeding twice daily with granulated food (Tetra granules, Germany) and once with Artemia sp. nauplii. One female and one male zebrafish were isolated in mating tanks the night before egg collection. After the lights were turned on in the morning, the separator was removed to allow fertilization.

### 2.2 Morpholino injection

Upon spawning, embryos were immediately collected and microinjected within 45 min, at the 1-2-cell stage, with 1nL of morpholino solution containing either 0.8pmol CTRLMO (control morpholino) or MCT8MO (*mct8* morpholino) as described (27). The diffusion process of the morpholino compound is immediate and ubiquitous throughout the embryo, and the blocking effect over *mct8* lasts robustly up to 72hpf.

Then, embryos were randomly distributed into plastic plates containing E3 medium (5 mM NaCl, 0.17 mM KCl, 0.33 mM CaCl, 0.33 mM MgSO4) and incubated until sampling time at 28.5°C (Sanyo, Germany) under 12h:12h light: dark cycles.

### 2.3 Analysis of gene expression

Embryos were manually dechorionated, snap-frozen in liquid nitrogen, and stored at -80°C. Embryo staging was performed by observing control embryos’ developmental landmarks (46). Eight independent biological replicates (pools of 20 embryos) were sampled at 10, 12, 18, 22, and 25 hpf (hours post fertilization), and eight biological replicates (pool of 15 embryos) were sampled at 30, 36, and 48hpf. Total RNA was extracted from the embryos with an OMEGA Total RNA extraction kit I (Omega Biotek, USA), followed by treatment with Ambion Turbo DNA-free kit (Life Sciences, USA), according to the manufacturer’s instructions. RNA concentration was determined by spectrophotometry using NanoDrop ND-1000 (NanoDrop Technologies Inc., USA), and integrity was determined by visualization in an agarose gel stained with SYBR Green (ThermoFisher Scientific). Only total RNA samples with a 2:1 ratio of 28s:18s rRNA were used in the analysis.

Synthesis of cDNA with 500ng of purified total RNA was reverse transcribed using RevertAid First Strand cDNA Synthesis and Random Hexamer Primers (Thermo Fisher Scientific, USA). cDNA was diluted 1/5 in ultrapure water and stored at -20°C. The quantification method used with the RT-QPCR method was the absolute quantification method, which determines the number of mRNA copies in the sample from a standard curve. Primers were designed using Primer 3 Plus using RNA-seq data (45). **Supplementary Table 1** provides primer sequences amplicon size and RefSeq for each gene included in the analysis. The gene’s target sequence was amplified by PCR, purified (EZNA Gel Extraction Kit, Omega Biotek), quantified (NanoDrop Technologies Inc., USA), and sequenced by Dye-termination to confirm identity. Quantitative real-time PCR (qPCR) was performed in a CFX-384 well (Biorad) with 6 µL of total volume. Final concentrations of PCR mix consisted of 1X SensiFASTTM SYBR, No-ROX Kit (Bioline, USA), 150nM forward primer, 150nM reverse primer, and 1 µL cDNA (1/5). The PCR amplification protocol was 95°C for 3 min, and 44 cycles of 95°C for 10 sec and 60°C for 15 sec, followed by a denaturation step from 60 to 95°C, 5 sec in 0.5°C increment, to obtain product specificity. Each cDNA sample was run as two technical replicates and averaged for expression analysis. Samples were discarded for quantification if the difference between replicates was over 0.5 cycles. No commonly used reference gene (*18S* and *gapdh*) presented invariable expression during the embryonic stages analyzed. Therefore, total RNA input was used as a normalizer according to the criteria for qPCR quantification in such cases (47).

### 2.4 Immunohistochemistry

One-cell stage embryos microinjected with either 0.8pmol of either CTRLMO (GeneTools) or MCT8MO (27) were fixed at selected stages in ice-cold 4%PFA/PBS overnight at 4°C. Samples were washed, depigmented when needed with PBS/0.3%H2O2/0.5%KOH, transferred into 100% methanol, and stored at -20°C until use. Samples in 100% MeOH were brought to room temperature and washed using a MeOH : PBS series (100% MeOH to 100% PBS). Embryos were hydrated, washed in PBS with 0.1% Triton X-100 (PBTr), and blocked with the addition of 10% sheep serum (Sigma-Aldrich Aldrich). Primary antibodies used were: 1:500 rabbit anti-HuC/D (16A11 - Invitrogen), 1:100 CF594 mouse anti-Zrf1 (ZDB-ATB-081002-46, ZIRC) and 1:50 mouse anti-Nkx6.1 (F55A10 DSHB). Samples were washed, and secondary antibody fluorescent labelling was carried out using 1:400 of goat anti-mouse IgG-CF594 (SAB4600321, Sigma-Aldrich), goat anti-rabbit IgG- Alexa 488 (111-545-047, Jackson Labs) or anti-mouse IgG-CF488 (SAB4600388, Sigma-Aldrich). Imaging was carried out in a Zeiss Z.1 light-sheet microscope. Images were imported into Fiji, and a region of interest was selected in a two-somite area (8800µm^2^) between somites 8-12. For neuron number determination, the 3D object counter in Fiji was used. Glial cell abundance was measured by determining the stained area after maximum intensity projection.

### 2.5 Riboprobe preparation and colorimetric whole-mount *in situ* hybridization (WISH)

Riboprobe synthesis, hybridization, and imaging of colorimetric *WISH* were performed as described in detail (27). To prepare *neurog1, fabp7a, slc1a2b, and olig2* riboprobes for *in-situ* hybridization, primers (**Supplementary Table 2**) were designed using as a template the sequences from the zebrafish assembled transcriptome (45). Analysis of cell distribution pattern (*her2*, *fabp7a*, *neurog1* and *slc1a2b*) on transverse sections of the spinal cord, *WISH* embryos were re-fixed in PFA 4%, dehydrated in MeOH/PBS and embedded in paraffin by isopropanol/paraffin gradient. Paraffin blocks were sectioned at 8µm and mounted on Poly-L-Lysine covered slides. Sections of interest were dewaxed, and coverslips were mounted with glycerol-gelatine (Sigma-Aldrich). Images of whole mounts and sections were acquired using a Leica LM2000 microscope coupled to a digital color camera DS480 (Leica).

### 2.6 Double fluorescent whole-mount *in situ* hybridization WISH

Riboprobes were generated as described in the previous section and labeled with either digoxigenin (Dig) (*mct8*, *thraa*, and *thrab*) or fluorescein (Fluo) (*her2*, *dla*, and *fabp7a*). A double hybridization procedure combining one Dig and one Fluo probe was performed in zebrafish embryos (10, 12, 18, 25, 30, 36, 48hpf) following (27). Antibody detection and development of the signal were carried out sequentially using a combination of antibody/Tyramide signal amplification (Perkin-Elmer, USA).WISH was carried out identically to (27) except hybridization was performed in the presence of 0.5 ng/mL of both Dig- and Fluo-labelled cRNA probes in HybMix. Stringency washes were performed as previously described (27). For the first probe detection, embryos were incubated overnight at 4°C in blocking solution MABTr/10% sheep serum (Sigma-Aldrich-Aldrich)/2% Blocking solution (Roche, Switzerland) with anti-DIG-POD Fab fragments serum (1:500, Roche, Switzerland). Embryos were washed in PBSTw and incubated for fluorescent color development in Alexa Fluor-594 Tyramide Reagent (ThermoFisher, USA), 1:100 in amplification reagent (Perkin Elmer), followed by several washes in PBSTw. To detect the second probe*, the* peroxidase activity of POD conjugated anti-serum was quenched by incubating samples for 1h in 3% H_2_O_2_ in PBS. Samples were washed in PBSTr and incubated overnight at 4°C MABTr/10% sheep serum (Sigma-Aldrich-Aldrich)/2% Blocking solution (Roche, Switzerland) with anti-Fluorescein-POD Fab fragments serum (1:500, Roche, Switzerland). Embryos were washed in PBSTw and then incubated in FITC-Tyramide (Perkin-Elmer) 1:100 in amplification reagent (Perkin Elmer), followed by several washes in PBSTw. Samples were stored in PBS containing 0.1% Dabco (CarlRoth, Germany).

### 2.7 Mitosis detection

Immediately after single fluorescent *WISH*, embryos were subject to immunohistochemistry to detect mitotic cells. The primary antibody used was rabbit anti-PH3 1:500 (06-570 Sigma-Aldrich-Aldrich), and the secondary antibody was goat anti-rabbit IgG-CF594 (SAB4600388, Sigma-Aldrich-Aldrich). Antibody incubation and blocking steps were performed in 1xPBS:10%Sheep serum.

### 2.8 Apoptosis detection (TUNEL assay)

Immediately after the single fluorescent *WISH* of *dla* and *her2*, cell death detection in embryos was determined by TUNEL assay using the *in-situ* cell death detection kit – TMR red (12156792910, Roche). According to the manufacturer’s instructions, including experimental controls. Briefly, samples were washed for 15 minutes at RT with 1xPBS/0.1% TritonX 100 (Sigma-Aldrich)/0.1 M Sodium Acetate pH6. Embryos were further treated for 15 minutes at RT with 1ug/mL Proteinase K (Sigma-Aldrich-Aldrich) followed by four 5 minutes of washes in 1xPBT.

### 2.9 Image acquisition and analysis of double *WISH*, *WISH*-mitosis and *WISH*-apoptosis

Light-sheet Z.1 (ZEISS) microscope was used to acquire images of double *WISH*, *WISH*-mitosis, and *WISH* apoptosis. Samples were mounted in 1% low melting agarose (CarlRoth, Germany). The total depth of the medial spinal cord was acquired using a 10x lens with 2.5x or 1x optical zoom, according to the developmental stage, using dual illumination and a z step of 1,69µm or 1,813 µm, according to the optical zoom in use. In addition, dual illumination image volumes from the Z.1 were merged by Dual Side Fusion (Zen Black, Zeiss), and imaging and colocalization analysis were performed in Fiji (48).

Colocalization Colormap plugin (49) was used to determine the colocalization of Dig and Fluo cRNA probes. Briefly, ROI was selected in a two-somite area (8800µm^2^) between somite 8-12. Next, the threshold was adjusted and fixed for each gene pair for the double WISH, and 3-8 individuals per condition were analyzed. The resulting stack of the colocalization channel was then superimposed into the original Z.1 image to create the final figures. When necessary, stacks were resliced in y orientation to enable lateral views.

For the *WISH*-mitosis and *WISH* apoptosis, the image threshold was adjusted and fixed for each target pair, and a total of 5-13 individuals per condition were analyzed. Colocalization Colormap plugin (49) was used to determine the colocalization of cRNA probes with mitotic marker PH3 and apoptotic marker TMR-red. Co-localized cells were counted manually with Fiji’s “3D object counter” tool.

### 2.10 NICD overexpression

In this experiment, a variant of zebrafish notch1a, notch1a-intracellular domain (NICD), was used, which encodes a Notch receptor that is constitutively active in neurogenesis (50). A pCS2+ plasmid containing the cDNA coding for CAAX-GFP (membrane label) and the Notch-intracellular domain (NICD) (50) were linearized, and mRNAs synthesized using the mMessage Machine SP6 transcription kit from Ambion, following the manufacturer’s instructions. The mRNAs were phenol: chloroform purified, diluted in RNAse-free water, and frozen at -80°C until use. The effects of this NICD mRNA injection are attributed to high NOTCH activity in general (50). The pCS2+ GFP-CaaX was generated after subcloning the GFP-CaaX construct from a Tol2 kit plasmid (51).

### 2.11 Live imaging

Zebrafish Tg(*elav3:LY-mCherry*) (52) x WT AB previously injected with CTRL or MCT8MO were used for mRNA injection. This transgenic line allows for the visualization of mature neurons, *elav3*, allowing us to distinguish them from neural progenitor cells. For mosaic overexpression of NICD, 100 pg of *nicd* mRNA and 50pg of *gfp-caax* mRNA were injected into one blastomere dorsal right 1 - DRA1 or dorsal right 2 -DRA2, between the 16- to 32-cell stages, which will contribute to brain and spinal cord cell fates (53). *gfp-caax* mRNA was used to allow the individual cell visualization of the cell-autonomous response to NICD in CTRL and MCT8MO injected embryos. Hence, four groups were prepared, GFP-CaaX injection in MCT8MO and CTRLMO; NICD and GFP-CaaX injection in MCT8MO and CTRLMO. Embryos were left to develop at 28°C until 22hpf when sorting and mounting for imaging were performed.

Imaging was carried out by light-sheet microscopy, Lightsheet Z.1 (ZEISS, Germany), as described previously (54), with minor alterations. Briefly, embryos were anesthetized with 0.08% tricaine pH7.4 buffered, mounted alive in 0.3% (w/v) low-melting agarose (LMA) in E3 medium containing tricaine (0.08%) into FEP tubes closed with a 1% LMA. Three animals per group, CTRLMO and MCT8MO, were imaged in the same tube. Two independent experiments were carried out. Time lapses images were taken from 23 until 26hpf. Z-stacks ranging from the full depth of the medial spinal cord were acquired every 15 min for 3h. The spinal cord was imaged with an x20 lens, 2x zoom with a z-step of 1.56 μm with single angle and dual illumination. For image analysis, dual illumination images from the Z.1 were merged using Dual side Fusion (Zen Black, Zeiss). Next, images were imported into Fiji, and a region of interest was selected in a two-somite area (8800µm^2^) between somite 8-12. Analysis of cell divisions was performed manually in FIJI. Only Huc(-) cells expressing GFP were tracked for analysis. In brief, symmetric divisions of GFP+ cells in any group were considered if the cell division plane was 0-<30° to the ventricular side of the spinal cord. Asymmetric divisions were considered if the cell division plane was ≥30-90° to the ventricular side of the spinal cord (55, 56).

### 2.12 Generation of Mct8 loss-of-function mutant

CRISPRScan (57) was used to design two adjacent guide RNAs (gRNAs) against the first exon of the zebrafish *mct8* gene (GGCTGGTGGGACGCCCGGCT and GGAGCGCAAGCTGGCCCCGG). gRNAs were purified after phenol-chloroform extraction and were precipitated overnight at -20°C in 10uL of 3.5M sodium acetate pH3.5 and 250uL of 100% ethanol. After centrifugation, gRNA was purified, dried, resuspended in DEPC-treated water, and kept at -80°C until use. The oligos for each gRNAs were acquired (STABvida) and used for direct *in vitro* transcription as described (57). On the injection day, the two gRNAs were diluted to 300ng/uL in a 600ng/uL Cas9 protein (Champalimaud Foundation) solution.

Adult zebrafish were made to spawn in natural conditions, and embryos were immediately collected. 1-cell stage embryos were used to inject 1nL of gRNAs+Cas9 (300ng/uL+600ng/uL). At 24hpf, eight embryos per injection clutch were collected for genotyping by PCR. Genomic DNA extraction was carried out after overnight digestion at 50°C in genomic extraction buffer (10mM Tris pH8.2, 10mM EDTA, 20mM NaCl, 0.5% SDS, 200ng/mL Proteinase K), followed by centrifugation and washing with 70% ethanol, air dried, and resuspended in 20uL of TE pH8. PCR was carried out with primers (0.2uM) flanking the gRNAs binding sites (Fw – ATGCACTCGGAAAGCGATGA; Rv – AGCAGCGAACACCACGACCCA) using the DreamTaq polymerase kit (Thermo). Thermocycling was carried out as follows: 95°C for 30 seconds, 35 cycles of 95°C for 30 seconds, 60°C for 15 seconds, and 72°C for 15 seconds, followed by a 5-minute extension at 72°C. PCR products were resolved in a 3.5% agarose/1xTAE gel. Afterwards, bands were isolated from the gel and extracted with a gel extraction band kit (OMEGA), followed by Sanger sequencing using the Big-dye termination method.

The isolated band sequence was confirmed after BLAST analysis and alignment to the zebrafish mct8 locus. That ensured that injected clutches had embryos carrying the desired genetic lesions on the mct8 locus. Injected embryos were reared until adulthood. After isolation, adult-inject PCR genotyped fish after fin-clipping to identify carriers of genetic lesions on the *mct8* locus. After sequencing, only carriers of mutations that induced an early STOP codon or a frameshift in the *mct8* ORF were allowed to cross with wild-type siblings to give rise to non-mosaic F1 carrier lines. Adult F1 carriers were genotyped by PCR after fin-clipping and sequenced. In-crosses were carried out to generate F2 homozygous mutants for the mct8 locus. Identified lines with embryos with expected phenotypes were collected, genomic DNA extracted, genotyped by PCR, and sequenced. Identified lines were crossed to wild-type siblings. Only F3 adult carriers were used to generate homozygous *mct8* mutant embryos. That was done to mitigate any possible non-specific genomic lesions other than in the *mct8* locus.

### 2.13 Statistical analysis

All statistical analyses were carried out in GraphPad Prism v6.01 (San Diego, USA). Values are represented as means ± SD. The datasets’ normality was previously accessed using D’Agostino & Pearson omnibus normality test. The levels of statistical significance were expressed as p-values, *p < 0.05; **p < 0.01; ***p < 0.001; ns: non-significant.

Due to the role played by the genes analyzed in embryonic development, the present work did not intend to determine their temporal expression patterns, only the effect of MCT8 knockdown on their expression at specific time points. To determine gene expression differences between CTRLMO and MCT8MO embryos, statistical significance was determined by unpaired Students t-test: two-sample, assuming equal variances. For image analysis quantification, One-way analysis of variance (ANOVA) followed by Dunnett’s multiple comparison tests or an unpaired Student’s t-test was used when data sets presented a normal distribution. Otherwise, a Kruskal-Wallis test followed by Dunn’s multiple comparison tests was used. Distribution differences in symmetric/asymmetric divisions between experimental groups were determined by χ2 analysis. Statistical difference in symmetric or asymmetric divisions between experimental groups was determined by one-way ANOVA followed by Holm-Sidak’s multiple comparison *post hoc* analysis.

## 3 Results

To further guarantee the validity of our MCT8 knockdown approach using morpholinos, its specific effects, and the lack of unspecific morpholino effects, we developed a CRISPR/Cas9 loss-of-function mct8 mutant (-/-) (**Supplementary Figure 1**). The mutant *mct8* (-/-) has an early STOP codon in the sixth codon, missense mutations, and a 9-bp insertion (**Supplementary Figures S1A, B**). After injection of the MCT8 morpholino at 0.8pmol in mct8 (-/-) embryos, we did not observe any additional effects different from control morpholino injected 24hpf mct8 (-/-) embryos (**Supplementary Figure 1C**). Complying with the best practices for using morpholinos (58, 59), our morpholino-based approach has shown to be highly specific and fully recapitulates the loss of function in the newly developed *mct8* (-/-) embryos without non-specific effects. Together with our previous validations (27, 45), these two converging models fully recapitulate the loss of MTH-impaired signaling during embryonic development.

### 3.1 Timing of MTH action in zebrafish embryogenesis

To determine the developmental time window of MTH action in zebrafish embryogenesis, we analyzed genes already known from previous transcriptomic data to have altered expression in 25hpf MCT8MO embryos (45) (**Supplementary Figure 2A**). Genes shown to be regulated by MTH involved in the early neural specification, NOTCH signaling pathway, and neurogenesis (**Supplementary Figure 2A**) were analyzed.

**Figure 1:**
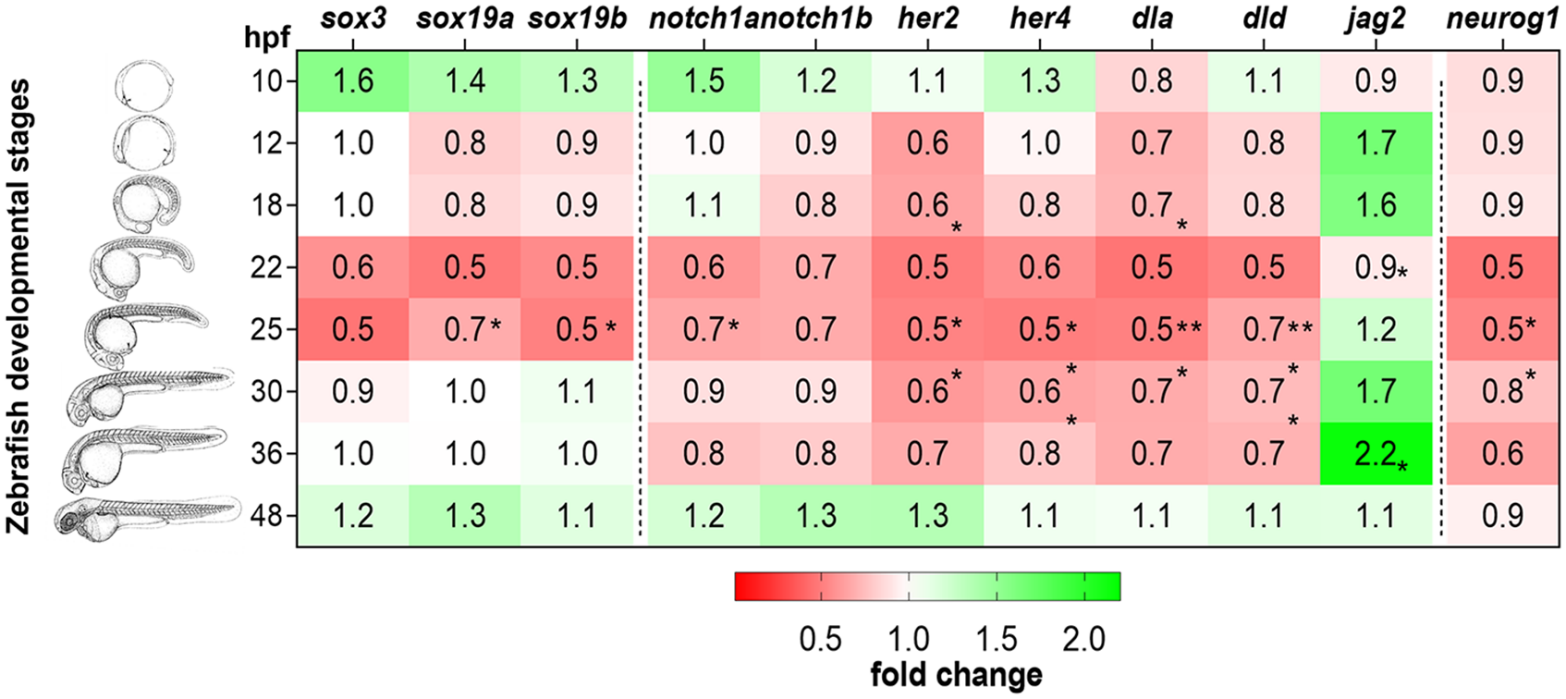
Expression of MTH-responsive genes reveals 22-31hpf as the developmental time more sensitive to MTH. Zebrafish developmental stages analyzed by qPCR are depicted by camera lucida drawings adapted from (46). Heatmap representation of gene expression levels of *sox3*, *sox19a*, *sox19b*, *notch1a*, *notch1b, her2*, *her4*, *dla*, *dld*, *jag2a*, and *neurog1*, determined after RT-qPCR in MCT8MO and CTRLMO during embryonic development. Data are represented as fold change of MCT8MO expression relative to the CTRLMO. Statistical differences were evaluated between MCT8MO and CTRLMO for each time point using a t-test after normal distribution was confirmed (D’Agostino & Pearson test). N = 8 (*p<0.05; **p<0.01).

Genes belonging to the SoxB1 family (*sox3*, *sox19a*, and *sox19b*) are recognized for their role in the specification and development of the embryonic ectoderm into the neuroectoderm lineage (60, 61). These candidate genes were downregulated at 25hpf in the MCT8MO RNA-seq data (**Supplementary Figure 2A**), indicating a possible role for MTH in maintaining the neuroectodermal progenitor pool. Analysis by qPCR (**Figure 1** and **Supplementary Figure 2B-D**) revealed that the expression of these genes did not change in MCT8MO embryos during early neurodevelopment (10hpf-18hpf). The results suggest that MTH does not play a role in maintaining B1 Sox gene expression during neural plate establishment and neural induction. However, expression of *sox19a* and *sox19b* is significantly lower in MCT8MO embryos at 22hpf (t-test, p<0.05), while *sox3* and *sox19b* show a decreased expression also at 25hpf (**Figure 1**), in accordance with RNA-seq data (45), although this change does not reach statistical significance in the qPCR assay (t-test, p=0.083, and p=0.079, respectively).

Notch ligand-receptor combinations that coincide during development in zebrafish are essential for adequate brain development and cell diversity (62). Gene expression analysis by qPCR revealed that only *notch1a* was significantly downregulated in MCT8MO at 22hpf (**Figure 1**; **Supplementary Figure 2E**; t-test, p<0.05) and continues to be lower than CTRLMO, in MCT8MO embryos until 36hpf, although not statistically significant (**Figure 1**; **Supplementary Figure 2E**). A similar trend occurs for the expression of the *notch1b* receptor; however, this decrease between 22-36hpf is not statistically significant (**Figure 1**; **Supplementary Figure 2F**, t-test, p>0.05).

The expression of NOTCH direct targets *her2* and *her4*, which are involved in the maintenance and proliferation of neuroprogenitor cells (63–65), was analyzed (**Figure 1**). Expression of *her2* is downregulated in MCT8MO embryos at 12, 22 and 25hpf (**Figure 1**; **Supplementary Figure 2G**, t-test, p<0.05).In zebrafish, *her4* is involved in primary neuron development under Notch 1 signaling (65). *Her4* downregulation at 22, 25, and 30hpf in MCT8MO suggests the involvement of MTH in regulating the development of some primary neurons (**Figure 1**; **Supplementary Figure 2H**, t-test, p<0.05).

Notch ligands *dla* and *dld*, which are expressed in differentiating neural cells and are involved in the specification of progenitor pool size domains (66), showed a significant decrease in expression in MCT8MO embryos at 25hpf (**Figure 1**; **Supplementary Figures 2I, J**, respectively, t-test, p<0.05). The downregulation of *dla* is observable by 12hpf (**Figure 1**; **Supplementary Figure 2I**, t-test, p<0.05) during primary neurogenesis and occurs at 22 and 25hpf (**Figure 1**; **Supplementary Figure 2I**, t-test, p<0.001 and p<0.05, respectively). On the other hand, the decrease in *dld* expression (**Figure 1**; **Supplementary Figure 2J**) only occurs later in neurogenesis at 22 (t-test, p<0.01), 25 (t-test, p<0.05), and 30hpf (t-test, p<0.05).

In contrast with *dla* and *dld*, the Notch ligand *jag2a* is upregulated in MCT8MO embryos (**Figure 1**; **Supplementary Figure 2K**, t-test, p<0.05). The temporal pattern of expression of *jag2a* was opposite to the delta ligands, *dla*, and *dld*, since it was upregulated at 18hpf (**Figure 1** p<0.05) and again at 36hpf (t-test, p<0.05).

To further understand how MTH is involved in neuron progenitor specification, we analyzed the expression of *neurog1*, a pro-neural gene expressed by intermediate neuronal precursors and neuron-committed cells. No differences in *neurog1* expression occur from 10-18hpf between CTRL and MCT8MO embryos suggesting MTH is not involved in the differentiation of these cells (**Figure 1** and **Figure 1** and **Supplementary Figure 2L**). However, at 22 and 25hpf (**Figure 1** and **Supplementary Figure 2L**, t-test, p<0.05), *neurog1* expression decreased, suggesting a possible role for MTH in the maintenance/differentiation of neuron progenitor populations from these stages of neurogenesis while no differences in expression were found at later stages (**Figure 1** and **Supplementary Figure 2L**, t-test, p>0.05).

### 3.2 Impaired MTH action has a time-dependent effect on spinal cord neural development

**Figure 2:**
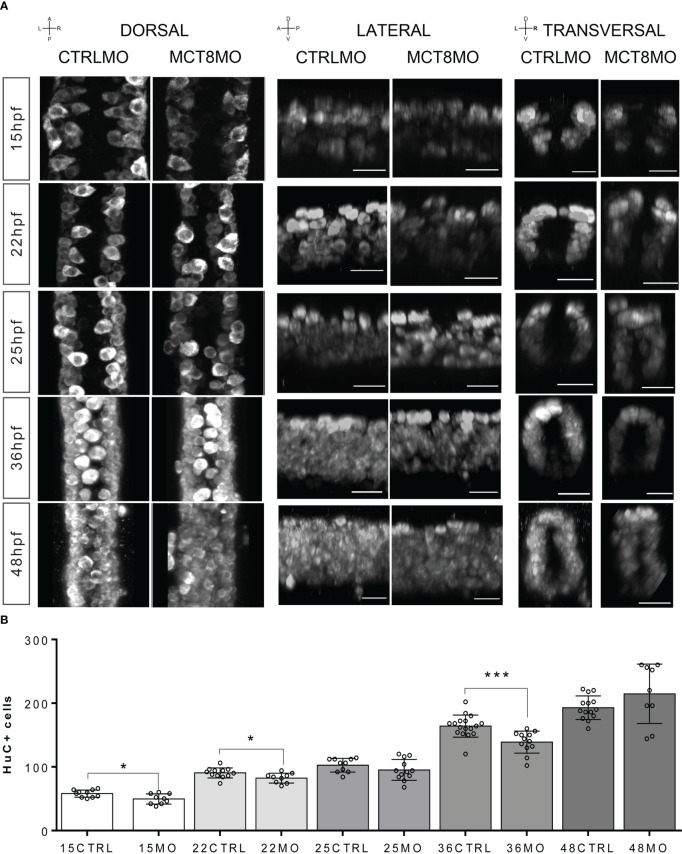
Compromised number and distribution of HuC/D neurons in MCT8MO embryos at specific stages of development. **(A)** Representative maximum projection images of the pan-neuronal marker HuC/D immunostaining (white) in the spinal cord between somite 8-12. Comparison of the pattern of neuron distribution in the spinal cord between CTRLMO and MCT8MO embryos at different stages of development. Red highlight - dorsal views, anterior spinal cord up. Blue highlight - lateral view, anterior spinal cord right. Green highlight -transversal view, dorsal spinal cord up. Scale bars represent 25 µm. **(B)** Quantification of the number of HuC/D single positive cells in a 2-myotome length of the spinal cord. n=9-17. CTRL (CTRLMO); MO (MCT8MO). Results are presented as mean ± SD; Statistical significance determined by t-test: two-sample, assuming equal variances: *p<0.05; ***p<0.001.

We interrogated if gene expression changes are paralleled with neurogenesis and gliogenesis changes, focusing on the spinal cord since it provides a simplified version of neural development. Immunostaining for Elav3 (HuC/D) of CTRLMO and MCT8MO zebrafish, which labels all post-mitotic neurons from 15-48hpf, revealed a time-dependent topology and abundance of neurons (**Figure 2**). Notably, in all developmental stages analyzed, the distribution of neurons in the three regions of the spinal cord (dorsal, medial, and ventral) present different HuC/D staining profiles between CTRLMO and MCT8MO embryos (**Figure 2A**). From as early as 15hpf, neurogenesis was impaired, as can be seen by the decrease in post-mitotic neurons in MCT8MO (**Figure 2B**; t-test, p<0.05). The most affected spinal cord neuron population in MCT8MO embryos is medial, as can be observed in lateral and transversal sections (**Figure 2A**). As development progresses, at 22hpf, there are fewer neurons, and the distribution is different in MCT8MO embryos (**Figures 2A**, B; t-test, p<0.05). That is especially evident in the lateral view, where medial and ventral neurons seem to be particularly affected. By 25hpf, and although neuron numbers have recovered (**Figure 2B**; t-test, p>0.05), MCT8MO neuron distribution is more compact (**Figure 2A** transversal view) with an apparent accumulation of dorsally located neurons, some of which appear to be out of the spinal cord scaffold (**Supplementary Figure 3**). The different distribution of the cells between CTRLMO and MCT8MO embryos is exacerbated at 36hpf, where dorsal neurons seem to increase with a simultaneous decrease in medial and ventral neurons. Additionally, at 36hpf, neurons are decreased in MCT8MO (**Figure 2**; t-test, p<0.0001). By 48hpf, the distribution of neurons in any view of the spinal cord is different in CTRLMO and MCT8MO embryos, especially evident dorso-ventrally (**Figure 2A** lateral and transversal views), but there is no difference in the neuron number (**Figure 2B**; t-test, p>0.05).

**Figure 3:**
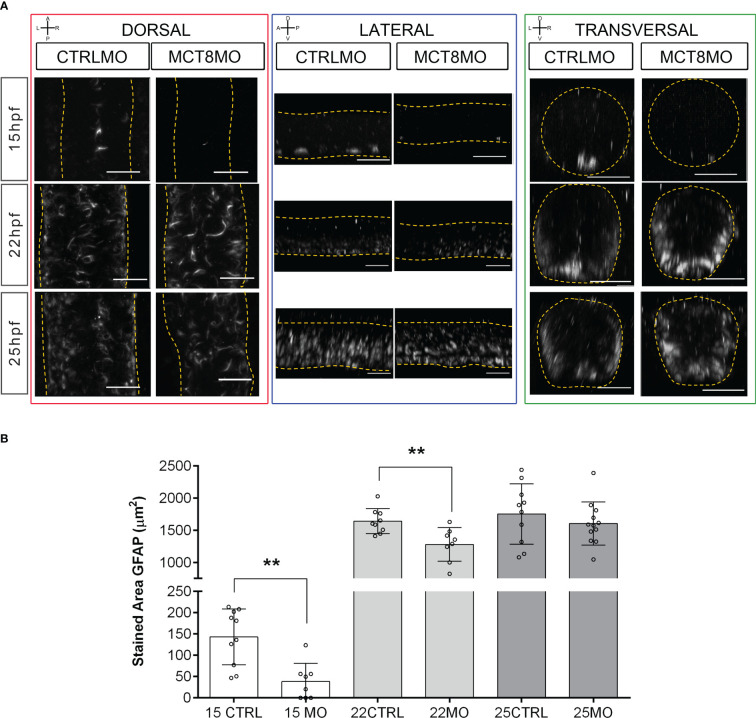
MCT8MO embryos have altered glial cell development during early neurogenesis. **(A)** Representative maximum intensity projection images of the spinal cord between somite 8-12 after glial cell labelling with ZRF-1 immunostaining (white, labelling GFAP fibers). In control embryos, at 15hpf glial cell fibers are organized in the developing ventral spinal cord; in MCT8MO embryos, the development of these cells is delayed, and only some scattered GFAP fibers are detected in the ventral-most neural tube. At 22hpf, the neural tube is closed, and glial cells can be detected throughout the spinal cord of CTRLMO and MCT8MO embryos; at 25hpf, the patterning of glial cells is altered in MCT8MO embryos. Red highlight - dorsal views, anterior spinal cord up. Blue highlight - lateral view, anterior spinal cord right. Green highlight - transversal view dorsal spinal cord up. All scale bars represent 25 µm. Dashed yellow lines denote spinal cord boundaries. **(B)** Quantification of the area of GFAP staining in a 2-myotome length of the spinal cord. n=9-17. CTRL (CTRLMO); MO (MCT8MO). Results are presented as mean ± SD; Statistical significance determined by t-test: two-sample, assuming equal variances: **p<0.01.

We also interrogated how spinal cord gliogenesis was affected by impaired MTH signaling (**Figure 3**). To this end, embryos were immunostained with an anti-GFAP serum, and the stained volume of a 2 myotome section of the spinal cord was determined. We observed a time-dependent effect of impaired MTH action on gliogenesis up to 25hpf in MCT8MO (**Figure 3**). At 15hpf, there is a noticeable reduction of GFAP staining in MCT8MO embryos (**Figure 3**, t-test, p<0.001) with a very restricted GFAP signal (**Figure 3A**). In contrast to CTRLMO embryos, in MCT8MO morphants, the ventral signal of GFAP at 15hpf was spread along the left-right axis of the spinal cord. In contrast, medial and dorsal staining was mostly lost (**Figure 3A**, transversal). By 22hpf, the topology of GFAP staining was different between groups (**Figure 3A**), and the overall stain by GFAP was lower in MCT8MO (**Figure 3B**, t-test, p<0.01). By this time, the GFAP signal in MCT8MO embryos increased in the lateral basal edge of the spinal cord, and little to no signal was found in the apical region. At 25hpf, the signal distribution in any axis differs between the two experimental groups (**Figure 3A**). In contrast, in CTRLMO, GFAP staining lined the basal edge of the spinal cord, while in MCT8MO embryos, it was scattered throughout the basal-apical orientation (**Figure 3**, transversal). However, the stained volume of GFAP in the spinal cord is similar in both groups (**Figure 3B**, t-test, p>0.05). The observation argues that MTH modulates gliogenesis, determining the position of glial cells and likely the cell diversity generated in this neural population.

**Figure 4:**
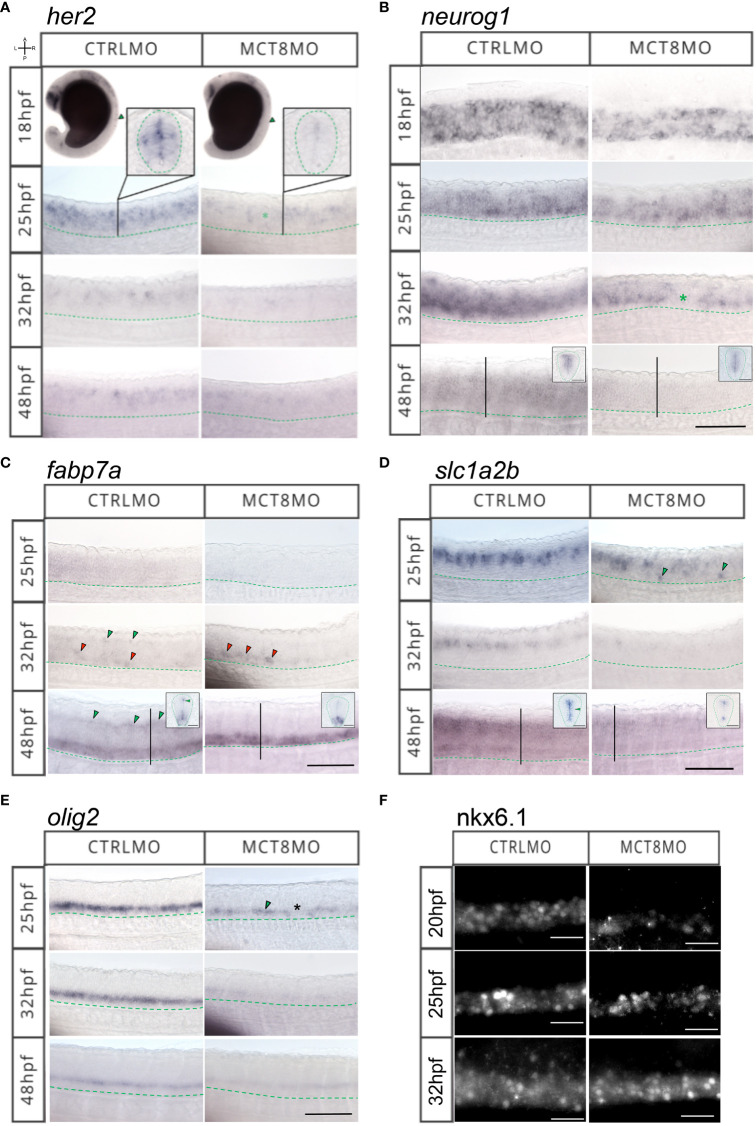
MTH is necessary for developing and correctly positioning neural cells in the spinal cord. WISH expression pattern of **(A)** *her2*, **(B)** *neurog1*, **(C)** *fabp7a*, **(D)** *slc1a2b*, **(E)** *olig2*, **(F)** IHC for Nkx6.1 in the spinal cord (SC) of zebrafish during embryonic development in MCT8MO and CTRLMO embryos. **(A)** Green asterisk in 25hpf MCT8MO represents absence of *her2* expression. **(B)** Green asterisk in 32hpf MCT8MO highlights absence of *neurog1* expression. **(C)** Green arrowheads at 32hpf CTRLMO, indicate the dorsal *fabp7a*+ cells which are lost in MCT8MO. Red arrowheads indicate increased *fabp7a* staining in the ventral domain in MCT8MO. At 48hpf, green arrowheads indicate *fabp7a*+ cells in the CTRLMO SC dorsal domain, which are less evident in MCT8MO. **(D)** At 25hpf, green arrowheads indicate misplaced *slc1a2b*+ cells in the ventral SC of MCT8MO. At 32hpf, the expression of *slc1a2b* is less abundant in MCT8MO. At 48hpf MCT8MO display reduced *slc1a2b* signal in cells at the most ventral and dorsal regions of the neurocoelom. The green arrowhead in the CTRLMO inset indicates an area where *slc1a2b* expression is less abundant in MCT8MO. **(E)** In CTRLMO, *olig2*+ cells were present in the most ventral region of the SC. At 25hpf, the green arrowhead indicates the position of *olig2*+ cell clusters, and the asterisk denotes the absence of cells in MCT8MO. **(F)** Nkx6.1 immunofluorescence detection at 20, 25, and 32hpf. A minimum of 10 individuals/conditions/gene or protein were analyzed. Images represent a lateral view of the SC (unless specified) between somites 8-12 with rostral orientation located to the left; Green dashed line represents the ventral limit of the SC; Scale bars represent 50 µm. Insets in **(B–D)** at 48hpf are transverse sections; Green dashed line represents the outermost boundary of the SC; Scale bars represent 20 µm.

To further dissect which cell populations in the spinal cord are affected by lack of MTH, we analyzed the expression of genes involved in neural progenitor specification (*her2*, **Figure 4A**), neuron committed progenitors (*neurog1*, **Figure 4B**), radial glial cells (*fabp7a*, **Figure 4C**), astrocyte-like cells (*slc1a2b*, **Figure 4D**), oligodendrocytes (*olig2*, **Figure 4E**) and motorneurons (Nkx6.1, **Figure 4F**).

*her2*+ neural progenitors are lost from as early as 18hpf in MCT8MO, and dorsal populations are most affected (**Figure 4A**). Cells expressing *her2* become more spaced, suggesting that some but not all *her2*+ progenitors are more susceptible to impaired MTH signaling than others (**Figure 4A**).

A similar situation is observed for *neurog1*+ neuron committed progenitors (**Figure 4B**). At 18hpf, there are significantly fewer *neurog1*+ cells in MCT8MO (**Figure 4B**), leading to a primarily complete loss of dorsal *neurog1*+ cells by 25hpf s (**Figure 4B**). This pattern continues at 32hpf. Additionally, gaps are observed in *neurog1*+ cell staining (green asterisk in **Figure 4B**). That observation suggests that specific *neurog1*+ progenitors at specific spinal cord locations depend more on MTH than others. By 48hpf, *neurog1*+ progenitors in MCT8MO embryos are restricted to the more ventricular region of the spinal cord surrounding the neurocoelom (inserts in **Figure 4B**).

At 25hpf *fabp7a*+ radial glial cells seem to be highly dependent on MTH for their development, given their almost absence in MCT8MO embryos (**Figure 4C**). By 32hpf, some *fabp7a*+ cells are found; however, these are primarily ventral (red arrowheads in **Figure 4C**). In contrast, dorsal *fabp7a*+ cells are lost (green arrowheads in CTRLMO embryos in **Figure 4C**). This effect is accentuated at 48hpf where no dorsal *fabp7a*+ cells (green arrowheads in CRTLMO embryos in **Figure 4C**) are found in MCT8MO embryos. However, the ventral expression field of *fabp7a*+ in MCT8MO embryos is more extensive and presents a different spatial distribution than in CTRLMO embryos (inserts in **Figure 4C**).

Astrocyte-like cells expressing *slc1a2b+* are also affected in MCT8MO embryos (**Figure 4D**). Already by 25hpf, there is a decrease in expression of *slc1a2b* in the dorsal spinal cord of MCT8MO embryos (**Figure 4D**) with a less dense row of cells present, while concomitantly with the development of ventral located *slc1a2b*+ cells (green arrowheads in **Figure 4D**) which are absent in control embryos. By 32hpf and 48hpf, there is a general decrease of *slc1a2b*+ cells in MCT8MO embryos’ spinal cord (**Figure 4D**), which at 48hpf is accompanied by a restriction of the expression field, which confines to the most dorsal and ventral regions of the spinal cord canal (inserts in **Figure 4D**).

In MCT8MO embryos, *olig2*+ cells in the spinal cord decrease from as early as 25hpf (**Figure 4E**). This reduction is even more apparent at 32hpf but slightly recovers by 48hpf (**Figure 4E**). Nonetheless, *olig2*+ staining is always lower in MCT8MO than in control embryos (**Figure 4E**), suggesting the loss of some cells.

The Nkx6.1+ motorneuron cells are strongly decreased in MCT8MO embryos as early as 20hpf. That is still noticeable at 25hpf, but at 32hpf, there is an expansion of the Nkx6.1+ domain in MCT8MO embryos that is broader than in control embryos (**Figure 4F**). Moreover, a medial to a dorsal expansion of Nkx6.1 cells occurs in MCT8MO embryos, while in control embryos these are primarily concentrated in a ventral position (**Figure 4F**), suggesting that the identity of these Nkx6.1+ cells may not be identical in CTRL and MCT8MO embryos.

### 3.3 MTH is essential for a subset of neural progenitor cells to survive and proliferate

The previous results suggest that MTH is involved in specifying distinct neural populations. The decrease in *her2, neurog1*, and *fabp7a* expression in *mct8* morphants suggest that the function of MTH in the generation of cell diversity in the zebrafish spinal cord arises already at the progenitor level, either by restricting the fate of daughter cells or restricting the diversity within the progenitor pool itself.

**Figure 5:**
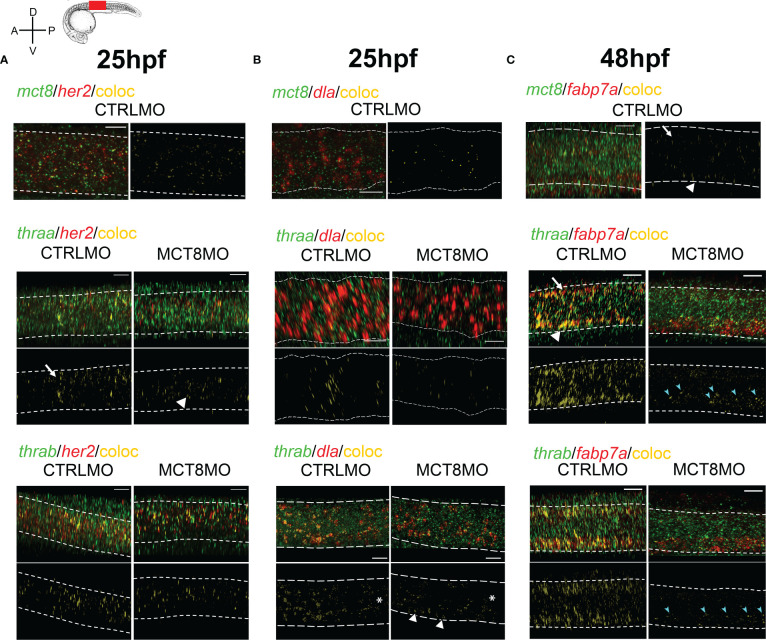
MTH is directly involved in the development of discrete *her2*, *dla*, and *fabp7a* cells. Colocalization of zebrafish *thraa*, *thrab*, and *mct8* with *her2* and *dla* expressing cells at after double WISH. *thraa*, *thrab* and *mct8* (green); *her2, dla* and *fabp7a* (red) and colocalization (yellow). **(A)** At 25hpf, *thraa*/*her2* colocalization in CTRLMO embryos (arrow) is increased in the apical spinal cord, while in MCT8MO *thraa*/*her2* colocalization has a more medial distribution in the spinal cord (arrowhead). **(B)** At 25hpf, *thrab*/*dla* colocalization is less predominant in MCT8MO embryos, and asterisks denote decreased colocalization along the anterior-posterior axis of the spinal cord. Arrowheads indicate the increased colocalization of *thrab/dla*+ in cells of the ventral spinal cord in MCT8MO embryos. **(C)** At 48hpf, *mct8/fabp7a* colocalization in CTRLMO occurs scattered through the spinal cord with an arrow indicating colocalization in the dorsal spinal cord and arrowhead colocalization in the ventral spinal cord. Colocalization of *fabp7a* with *thraa* and *thrab* in MCT8MO embryos is more restricted to the ventrally located *fabp7a+* cells (blue arrowheads). All images depict a spinal cord section between somite 8-12; rostral is left and dorsal up. White dashed lines show the boundary of the spinal cord. A minimum of 3 individuals/conditions was analyzed. The scale bar represents 20µm.

All components of T3 cellular signaling (i.e. *mct8*, *thraa*, and *thrab*) are already present in the zebrafish neuro-epithelium from as early as 12hpf and widely expressed in the spinal cord up until 48hpf (**Supplementary Figure 4**). At 25hpf, several MTH sensitive (*mct8+*) *her2+* neural progenitors are present in a scattered pattern more frequently in the ventral half of the spinal cord (**Figure 5A**). In a receptor-specific pattern, *thraa* is mostly co-expressed with *her2* dorsally (arrow) and continuously expressed anterior-posteriorly (**Figure 5A**). *thrab/her2* co-expression also appears anterior-posteriorly; however, it is present in bands separated by regions of no co-expression (**Figure 5A**). These *thrab/her2* co-expression bands spawn the dorsal-ventral axis but are more frequent in the medial region of the spinal cord. In MCT8MO embryos, co-expression of *her2* with the receptors is not lost. However, it decreases and presents different distributions (**Figure 5A**). In MCT8MO embryos, *thraa/her2* co-expression becomes more ventral (arrowhead) and medial, even though some dorsal co-expression is visible (**Figure 5A**). *thrab/her2* co-expression loses the anterior-posterior pattern of defined bands, becoming continuous and more restricted to the medial region of the spinal cord (**Figure 5A**).

These findings indicate the existence of at least six *her2*+ neural populations dependent at some point on MTH signaling components during spinal cord development: MTH+/*thraa*/+*thrab*+, MTH+*/thraa*+/*thrab*-, MTH+/*thr*aa-/*thrab*+, MTH-/*thraa*+/*thrab*+, MTH-/*thraa*+/*thrab*- and MTH-/*thraa*-/*thrab*+.

At 25hpf, MTH sensitive cells (*mct8+*) *dla+* cells are restricted to the medial region of the spinal cord. No *mct8/dla* co-expressing cells are detected in the spinal cord’s most dorsal and ventral regions (**Figure 5B**). At this time, spinal cord *thraa/dla* co-expression has a very defined anterior-posterior expression pattern in bands that spawns dorso-ventrally but is more frequent medially (**Figure 5B**). In contrast, *thrab/dla* co-expression has an anterior-posterior decrease in frequency (asterisks) but is uniformly distributed dorso-ventrally and in large clusters (**Figure 5B**). At 25hpf, *mct8* morphant embryos’ co-expression of *dla* with *thraa* or *thrab* is severely decreased, and its distribution changed compared to control siblings (**Figure 5B**). In these embryos, *thraa/dla* colocalization loses the pattern found in control siblings and is scattered with some larger clusters found in discrete dorsal, medial and ventral regions of the spinal cord (**Figure 5B**). In turn, *thrab/dla* colocalization still presents a decreased anterior-posterior expression (asterisk) but is almost lost dorsally and medially (**Figure 5B**). Although decreased compared to CTRLMO embryos, the co-expression of *thrab/dla* is more frequent at a ventral position (arrowheads in **Figure 5B**). These observations indicate that only a fraction of the *dla+* cells depend on MTH, since most *thraa+/dla+* co-expression is lost in MCT8MO. In the MTH-sensitive, *mct8+*/*dla+* population cells, *thraa* is likely the primary receptor mediating the genomic action of the hormone. In MCT8MO a similar situation occurs for *thrab+/dla+* co-expression, although not so widespread. Moreover, comparing the co-expression patterns between groups indicates that: i) in most dorsal and ventral regions of the spinal cord, *thrab/dla+* cells are likely mostly irresponsive to MTH and ii) most MTH sensitive *thrab+/dla+* cells have a medial localization (**Figure 5B**).

We further looked at *fabp7a* colocalization with *mct8*, *thraa*, and *thrab* at 48hpf, a time of extreme sensitivity of radial glial cells (RGC) to MTH (**Figure 4C**). In CTRLMO, colocalization of *mct8+/fabp7a+*, is primarily located on the most dorsal (arrow) and ventral (arrowhead) regions of the spinal cord (**Figure 5C**). Most *fabp7a*+ cells co-express *thraa* and or *thrab* (**Figure 5C**), indicating that RGCs are highly dependent on MTH regulated transcription. That becomes even more evident when we look at MCT8MO embryos, where there is a drastic decrease in the frequency of *fabp7*a co-expression with *thraa* and *thrab* (**Figure 5C**). In fact, dorsal co-expression of *fabp7a* and thrab is almost entirely lost (**Figure 5C**). Ventrally, in the MCT8MO, a different scenario is found. Although most co-expression of *fabp7a* with *thraa* and *thrab* is lost, *fabp7a* expression increases (**Figure 5C**). Notably, the remaining co-expression fields of *fabp7a* with either *thraa* or *thrab* are in clusters on the dorsal portion of the most ventral third of the spinal cord (cyan arrows in **Figure 5C**). Nonetheless, the superimposition of the two co-expression fields does not retire the possibility that in some *fabp7a+* cells, MTH action occurs *via* both receptors (**Figure 5C**). Together these observations indicate that: i) dorsal developing *fabp7a+* RGC rely more on MTH to differentiate than ventral RGC; ii) ventrally MTH action might be more important in RGC fate decisions and diversity (specialization) generation than general RGC *fabp7a+* development; iii) most *fabp7a+* RGC are dependent on MTH genomic action, but a small portion of RGC depend on thyroid receptor aporeceptor function to develop.

**Figure 6:**
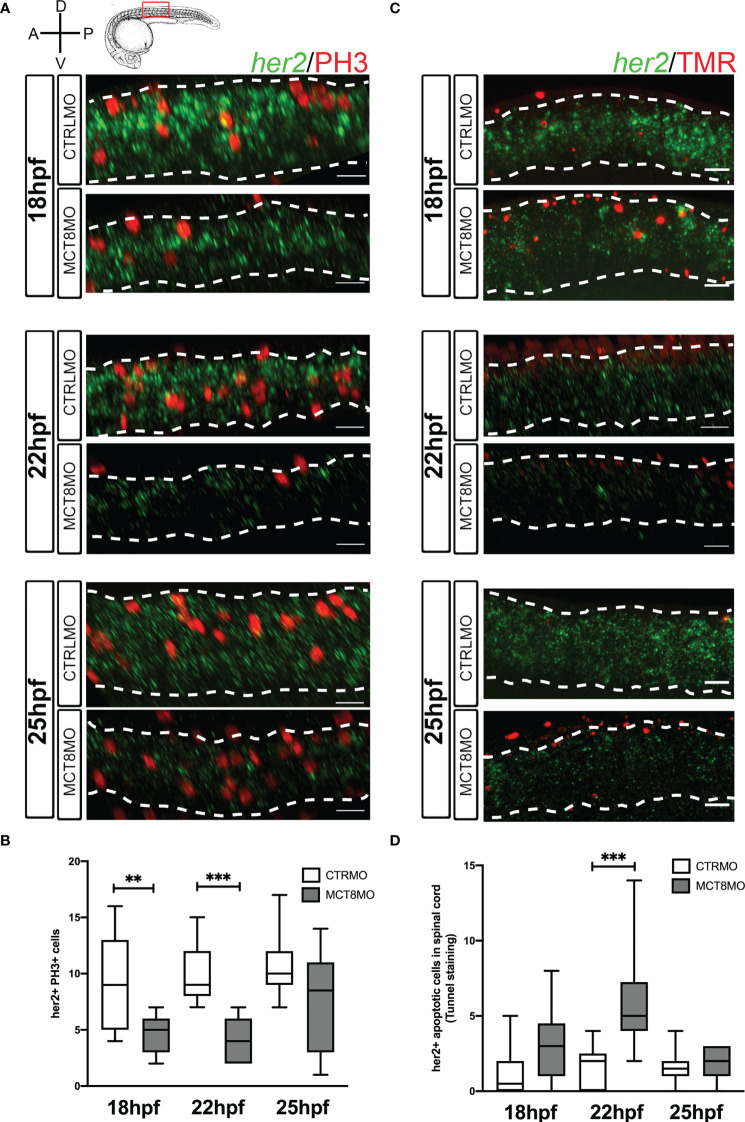
Impaired MTH signaling decreases *her2+* neural progenitor cells undergoing mitosis in the spinal cord. **(A)** Analysis of *her2* expression by fluorescent *in situ* hybridization (green) and mitotic cells (phosphohistone 3 antibody; red) in CTRLMO) and MCT8MO embryos. **(B)** Box-and-whiskers plot depicting quantification of the number of *her2*+ mitotic cells (*her2*+/PH3+) in the spinal cord at 18, 22, and 25hpf. **(C)** Analysis of *her2* expression by fluorescent *in situ* hybridization (green) and colocalization with apoptotic cell detected using a TUNEL assay (red). **(D)** Box-and-whiskers plot depicting the quantification of the number of *her2*+ apoptotic cells in the spinal cord. The images represent a lateral view of the spinal cord between somite 8-12; rostral is to the left in all images; the scale bar represents 50 µm. Colocalization was quantified in the volume of 2 myotomes within this spinal cord region. n=10-15. Statistical significance determined by t-test: two-sample, assuming equal variances. **p<0.01; *** p<0.001.

**Figure 7:**
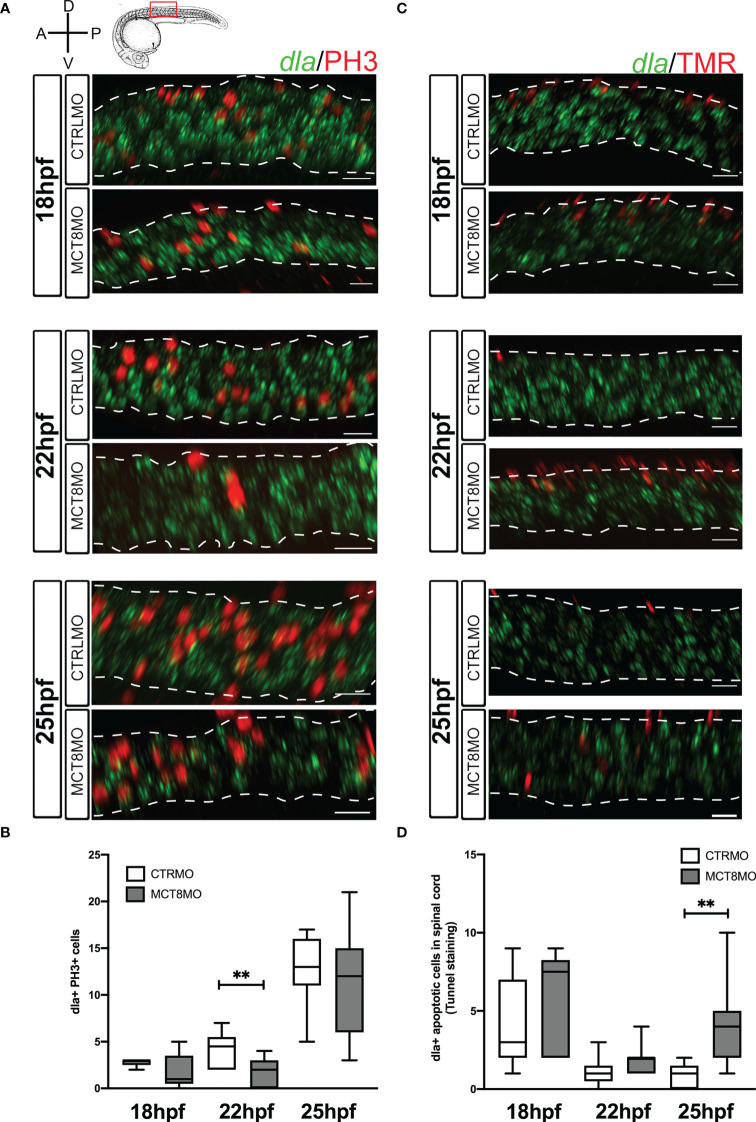
Impaired MTH signaling affects proliferation and apoptosis of *dla+* spinal cord cells in a time-restricted manner. **(A)** Analysis of *dla* expression by fluorescent *in situ* hybridization (green) and colocalization with cell mitosis (phosphohistone 3 immunostaining, red) in CTRLMO) andMCT8MO embryos. **(B)** – Box-and-whiskers plot of quantification of the number of *dla*+ mitotic cells in the spinal cord at 18, 22, and 25hpf in control and MCT8MO injected embryos. **(C)** Analysis of *dla* expression by fluorescent *in situ* hybridization (green) and colocalization with apoptotic cell detected using a TUNEL assay (red). **(D)** Box-and-whiskers plot quantifying the number of *dla*+ apoptotic cells in the spinal cord at 18, 22, and 25hpf in control and MCT8MO injected embryos. The images present a lateral view of the spinal cord between somite 8-12, rostral is to the left, and dorsal up in all images. The scale bar represents 50 µm. Colocalization was analyzed in 2 myotome volume sections within this spinal cord region (n=10-15). Statistical significance determined by t-test: two-sample, assuming equal variances. **p<0.01.

To further understand how MTH acts on spinal cord *her2+* and *dla+* neural progenitors’ development, we carried out assays to understand if these cells stop proliferating or undergo apoptosis when MTH uptake by MCT8 is blocked (**Figures 6**, **7**). We analyzed embryos between 18hpf and 25hpf when qPCR analysis showed that *her2* and *dla* expression was more responsive to MTH (**Figure 1**). In general, cell proliferation (as measured from mitotic index labeling PH3) is decreased by ~50% on average at 18 to 25hpf in MCT8MO (**Supplementary Figure 5A**, p<0.0001). On the other hand, in all developmental stages analyzed, apoptosis is increased 2-fold in MCT8MO relative to CTRLMO embryos (**Supplementary Figure 5B**, p<0.001). As previously reported (27), this increase in apoptosis is specific to the lack of MTH and cannot be rescued by p53 signaling abrogation, thus indicating a specific effect of impaired MTH.

At 18 and 22hpf, *her2*+ mitotic cells decreased ~50% in MCT8MO embryos (**Figures 6A, B**, p<0.01; **Supplementary Figures 6A-a**”). At 25hpf, there are no differences between the two groups. These results parallel the data obtained for general PH3 staining and suggest that about one-quarter of *her2+* mitotic cells depend on MTH to proliferate (**Figure 6B**; **Supplementary Figure 5A**). Loss of *her2+* mitotic cells in *mct8* morphants occurs more frequently in medium and ventral regions of the spinal cord at 18 and 22hpf (**Figure 6A**). By 25hpf, there is an evident increase in *her2*+ mitotic cells in these regions of the spinal cord in *mct8* morphants (**Figure 6A**; **Supplementary Figures 6A-a**”).

Apoptosis of *her2+* cells in MCT8MO is only higher at 22hpf (p<0.001) but not at 18 and 25hpf (**Figures 6C, D**; **Supplementary Figure 6B-b**”). The divergence of *her2+* apoptotic cells from general spinal cord apoptosis indicates that only a small subset of *her2+* arising at 22hpf are likely dependent on MTH to develop. Irrespective of any experimental group, apoptotic *her2+* cells are more frequent dorsally, especially at 22 and 25hpf (**Figure 6C**). Together, these observations indicate that from 18 until 22hpf, about one-quarter of *her2+* progenitors depend on MTH to survive. The evidence argues that the major role of MTH on *her2+* progenitors is likely involved in cell fate decisions and cellular diversity generation.

The proliferation of *dla+* cells depends on MTH only at 22hpf (**Figures 7A, B**, p<0.01). At that time, only one-sixth of *dla+* cells are proliferating; of these, only half seem dependent on MTH (**Figure 7B**; **Supplementary Figure 7A-a**”). Notably, *dla+* proliferating cells do not follow the same frequency observed for general proliferation in the spinal cord for CTRLMO and MCT8MO embryos (**Figures 7A, B**; **Supplementary Figure 5A**). At 22hpf, dla+ MTH-dependent proliferating cells in control embryos are mostly ventrally localized and mostly lost in the MCT8MO (**Figure 7A**).

In contrast to cell proliferation, *dla+* apoptotic cells in the MCT8MO are increased only at 25hpf (**Figures 7C, D**, p<0.01; **Supplementary Figure 7B-b**”), accounting for twice as much as those found in control embryos. Moreover, *dla+* apoptotic cells in control embryos only represent about 20% of all apoptotic cells in the spinal cord at 25hpf, thus suggesting that in MCT8MO, apoptotic *dla+* cells might represent a different *dla+* population than the one found in control siblings (**Figure 7D** and **Supplementary Figure 5B**). Notably, *dla+* cell death does not follow the same distribution for overall spinal cord apoptosis (**Figure 7D** and **Supplementary Figure 5B**). In both control and MCT8MO embryos, most *dla+* cells are found in the most dorsal region of the spinal cord at 18 and 22hpf (**Figure 7C**). In contrast, to control embryos, at 25hpf in *mct8* morphants, *dla*+ apoptotic cells locate mainly in the medial and ventral regions of the spinal cord. The results indicate that only a small subset of *dla*+ cells depend on MTH for proliferation and survival. Moreover, this dependence seems restricted to dorsally located cells and well-defined developmental times (**Figure 7**).

**Figure 8:**
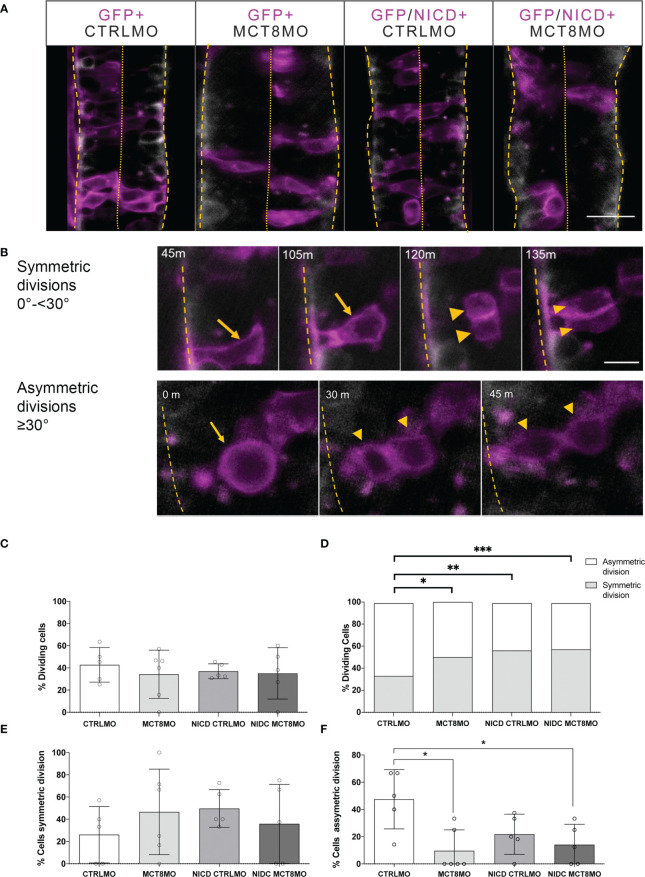
Effect of impaired MTH signaling and NICD overexpression on progenitor cell division. **(A)** At the start of imaging, representative images of the spinal cord of experimental Tg(elav3:LY-mCherry) embryos at 23hpf. Neurons (mCherry) are shown in white. Embryos were injected at the one-cell stage with either CTRLMO or MCT8MO followed by injection at the 16-cell stage in one blastomere with *gfp* mRNA only or *nicd* and *gfp* mRNA. Cells overexpressing NICD (and GFP) are labelled in magenta. Dorsal views of single slices between somite 8-15 are shown, anterior spinal cord up. The scale bar represents 50 µm. **(B)** Upper panel: Detail of symmetric division originating 2 morphologically similar GFP+ cells. A cell undergoing symmetric mitosis (yellow arrow), and the originating daughter cells (yellow arrowheads); Lower panel: Representative images of asymmetric division. A dividing cell (yellow arrow) originates two daughter cells (yellow arrowheads). Scale bar in B represent 20 µm. In all panels, the spinal cord basal limit is indicated by a dashed yellow line and the pial limit by dotted yellow lines. **(C–F) (C)** Percentage of analyzed GFP+ cells that underwent division. **(D)** Distribution of GFP+ dividing cells relative to all GFP+ cells observed in the period from 23-26hpf. χ2 analysis showed differences in the distribution of the number of cells undergoing symmetric or asymmetric divisions amongst experimental groups and CTRLMO (p<0.05). **(E)** Percentage of a cell undergoing symmetric division. **(F)** Percentage of cells undergoing asymmetric division. The results in C, D, and F are presented as the mean ± SD; results in D depict the ratio of cell division type in all GFP+ dividing cells analyzed. N =5-6 individuals per group (number of cells evaluated by group: CTRLMO/GFP=56; MCT8MO/GFP=77; CTRLMO/NICD/GFP=93; MCT8MO/NICD/GFP=46); Statistical significance in **(C, E, F)** was determined by a one-way ANOVA followed by a Holm-Sidak’s multiple comparison *post hoc* analysis, *p<0.05, **p<0.01, ***p<0.001.

The previous evidence further supports that NOTCH signaling mediates MTH action in zebrafish spinal cord neural progenitor cells. Furthermore, our evidence supports that the dependence of NOTCH signaling on MTH is highest between 18-30hpf. To further understand if this action of MTH can be cell-autonomously rescued by activated NOTCH signaling, each morpholino group was injected with either GFP mRNA or NICD+GFP mRNA, live imaging of the spinal cord was carried out between 23-26hpf (**Figure 8A**) and quantified symmetric and asymmetric GFP+ cells divisions in that period (**Figures 8B–F**). The NICD construct will activate the Notch signaling (50). No differences in the overall cell division of GFP-expressing cells between any of the experimental groups were observed (**Figure 8C**). However, there were significant differences in the proportion of symmetric/asymmetric divisions in control embryos with other experimental groups (**Figure 8D**; χ2, p ≤ 0.05). Nonetheless, symmetric divisions occurred more frequently in NICD+CTRLMO, MCT8MO, and NICD+MCT8MO experimental groups, although these were not statistically significant from the CTRLMO (**Figure 8E**, t-test p>0.05). In contrast, asymmetric divisions in MCT8MO and NICD+MCT8MO experimental groups were significantly less frequent compared to the CTRLMO group (**Figure 8F**, One-way ANOVA p<0.01, Sidak, p<0.05) but not the NICD+CTRLMO or between themselves. These results argue that NOTCH overexpression cannot rescue the lack of MTH signaling in these progenitor cells in a cell-autonomous manner.

## 4 Discussion

In the present work, we use an established zebrafish AHDS, MCT8 knockdown model and provide further evidence of a critical developmental time of MTH action. We reveal that in zebrafish, the period between 18-30hpf (~pharyngeal stage) is the most dependent on MTH based on the T3-responsive gene expression. This zebrafish developmental period corresponds to 8-24 weeks of human gestation, where MTH action in human embryonic development is essential for neurodevelopment (2, 3). Together, this data establishes a parallel action of MTH on neurodevelopment in humans and zebrafish.

Our analysis strongly supports that MTH action on target cells depends on tissue/cellular context. In zebrafish, as in mammalian systems, T3 is involved in the differentiation and proliferation of a wide variety of cell types, and this action depends on the cell identity, developmental state, and cellular context (27, 45, 67).

The data argue that MTH is not involved in neuroectoderm induction *via* B1Sox genes. However, at 12hpf, there a decrease in expression of Notch ligands *dla* and *her2* in the *mct8* morphants is observed, pointing out that a subset of neural progenitor cells already responds to MTH early in neurodevelopment. The identity of these early MTH-responsive cells and their progeny remains to be confirmed.

We demonstrate that MTH is essential for the proliferation and survival of both neural stem cells, *her 2+*, and committed neuron and glial progenitors, *dla*+. That argues that MTH regulation of neural diversity is likely achieved by modulation of the output from various progenitor cells. Inhibition of MTH uptake *via* mct8 transporter during zebrafish spinal cord neurodevelopment mainly affects the expression of dorsal *her2* + neural stem cells, *neurog1*+ intermediate neuron progenitors, *fabp7a+* and *pax6a+* (45) radial glial progenitors, and *olig2+* motoneuron and oligodendrocyte progenitor cells. Cells arising from these progenitors, such as *slc1a2+* astrocyte-like cells, Nkx6.1+ motoneurons, and *gad1b+* inhibitory interneurons (45), also show restricted development in their dorsal domains. The action of T3 on neuron development and survival has been described in chickens where mct8 knockdown leads to impaired optic tectum development, depletion of neuroprogenitors, and impaired neurogenesis with reduced neuron numbers and diversity (68). In *in vitro* mammalian cells, T3 is directly involved in the development of granule neurons by, on the one hand affecting the survival and differentiation of these cells (69) but also by preventing their apoptosis (70). Impaired maternal thyroid hormone signaling during mammalian neurodevelopment caused by mutations in thyroid hormone receptors giving resistance to T3 (71–74), congenital hypothyroid athyroid pax8 mutants, or double-knockdown *Mct8*/*Oatp1c1 (* 40, 75, 76), present similar cellular effects to the ones observed in zebrafish *mct8* morphant embryos ( (27, 45), present study). In the developing cortex of *Mct8*/*Octp1c1* double-KO embryonic mice (40, 41, 76), *Hr* and *gad67* (respectively, homologs of zebrafish *her2* and *gad1b*) expressing cells are mostly lost in the dorsal region, suggesting that in vertebrates MTH is an essential factor for dorsal specification of neuronal cell identities. Most notably, inhibitory neuron development seems particularly dependent on MTH action in mice (41, 76) and zebrafish (27, 45). In rat embryos, T3 deficiency decreases the proliferation and delays the maturation of the precursors of cerebellar GABAergic interneurons, with effects on the number of mature GABAergic neurons and GABAergic terminals (76, 77). A similar situation is found in a new mice AHDS model. Here a human AHDS-related mutation was introduced in the *Mct8* gene (P253L) and presented altered neuroarchitecture and impaired GABAergic neuron development, but no TH-target genes expression change is found at P90 (44). Notably, in zebrafish *mct8* morphants, a decrease in dorsal spinal cord neurons was observed simultaneously with an increase in ventral motoneurons (27). These suggest that the increase in excitatory neurons and depletion of GABAnergic interneurons contribute to the cellular basis of impaired locomotion observed in *mct8* morphants (27, 28) and human AHDS patients (78–80). Above all, a key observation in zebrafish *mct8* morphants is the recovery of spinal cord neuron numbers at 25 and 48hpf. However, these neurons’ identity, topology, and morphology are not identical to control morphants. That indicates that other neuron types assume their positions/locations in the loss of MTH-dependent neuron development, thus anticipating a compensatory mechanism. A similar compensatory mechanism was observed in Xenopus neurodevelopment. Here impaired NOTCH signaling leads to delayed neurogenesis, which is later compensated at the expense of impaired cell diversity (81). In human AHDS patients, microcephaly is rarely observed (79), strongly suggesting that impaired development of some neuron types leads to overgrowth from other types. The increased distribution domain of Nkx6.1+ neurons in MCT8MO suggests an alternate nature of Nkx6.1+ neurons. Indeed, in chicken embryonic retinal development, mct8 knockdown leads to a shift towards increased blue cones at the expense of green/red cones (82), confirming that in vertebrates, MTH is involved in generating neural cell diversity and the adequate balance between neuron types, in order to develop a fully functional central nervous system. In the adult mouse cortex SVZ, a similar role for T3 was found and mediated by TRa1,where the hormone balances the maintenance of the neurogenic progenitor pool and neuron differentiation (83).

The cellular mechanisms of TH action during neurodevelopment were also approached by determining the expression of TH machinery in neural spinal cord cells. In CTRLMO zebrafish embryos, co-expression of *her2* with *mct8* resembles mostly *her2* co-expression with *thraa*, suggesting that in *her2+* progenitors, effectuation of MTH signaling is *thraa* driven. Indeed, in *mct8* morphants, *thraa* co-expression with *her2* is mainly lost in dorsal spinal cord cells, whereas it is maintained chiefly ventrally. That argues that *her2+* dorsal NSC populations depend on MTH action *via* thraa, whereas ventral populations rely on thraa unliganded aporeceptor function to differentiate. A similar but not predominant situation appears with *thrab* since medial spinal cord co-expression with *her2+* is mainly maintained in *mct8* morphants but lost dorsally. From our analysis, the loss of dorsal *her2+* MTH-dependent progenitors is likely due to apoptosis since TUNEL staining strongly co-localizes with *her2+* cells in *mct8* morphants. A similar situation is found in the embryonic mouse cortex, where impaired MTH supply leads to decreased cell cycle length and apoptosis of progenitor cells (9, 84, 85). Moreover, in cultured rat pituitary tumor granule cells, T3-induced cell proliferation is mediated by changes in G1 cyclin/cyclin-dependent kinase levels and activity (86).

Previous transcriptomic analysis in zebrafish *mct8* morphants shows a steep decrease in the expression of cell-cycle genes (45), further strengthening this possibility. However, from our analysis, one cannot discard that decreased *her2+*MTH-dependent cells diminished their numbers after reduced proliferation due to precocious differentiation and exit from the cell cycle. Therefore, another possibility is that the lack of MTH leads these progenitors into senescence. That was previously observed in the neural stem cells of adult *Mct8*/*Octp1c1* double-KO mice mutants (87).

Our data also suggest that different progenitor populations respond to MTH differently. Although co-expression of *her2* and *dla* was previously observed by single-cell analysis in wild-type zebrafish embryos (88), the effect of MTH absence on proliferation and apoptosis of *her2* and *dla* expressing cells is unequal. *dla* is expressed in neural precursors and transiently in post-mitotic neurons at 11.5hpf (89). The increased cell death of progenitor cells, especially at an early stage of neurogenesis, can contribute to reducing progenitor pools leading to compromised neurogenesis. That is the case for oligodendrocyte progenitor populations in the zebrafish spinal cord development (90). In the case of *dla+* cells, regulation by MTH seems to depend on two different mechanisms where *thraa* and *thrab* have different roles in different spinal cord locations, suggesting the existence of at least three different *dla+* populations: one dependent on MTH and relies on *thraa*, one dorsal *thrab+* population dependent on MTH, and a ventral population that is positive to *thrab* but likely irresponsive to MTH. Nonetheless, and a limitation of this study, it is not yet possible to determine the identity and the progeny arising from these different *dla+* cell populations, and cell lineage studies are required to elucidate this aspect fully.

Interestingly, our data suggest that in zebrafish development, *fabp7a+* radial glial cells are highly dependent on MTH and that both *trhaa* and *thrab* are fundamental for the response of these cells to MTH. A similar situation occurs in the developing mouse hippocampus and cerebellum, wherein the hypothyroid embryo’s GFAP expression was markedly reduced in a time-dependent manner (91). Again, in MCT8MO, dorsal localized *fabp7a+* RGC are almost entirely lost while an expansion of *fabp7a+* RGC cells in the ventral domain occurs. In this ventral *fabp7a+* domain, colocalization with *thraa* or *thrab* is maintained in MCT8MO. Interestingly, in *mct8* morphants, *pax6a+* is also lost dorsally but less ventrally (45), further arguing for a differential dorsal-ventral role of MTH in RGCs development. From these observations, MTH is involved in the specification of different *fabp7a+* RGCs in the spinal cord, which is then reflected in the restricted development of *slc1a2b+* astrocyte-like cells in *mct8* morphant embryos. Our data indicate that MTH is essential to establish the correct combination of glial cell types that allow the development of adequate cytoarchitecture of the spinal cord. The observation further supports the finding that in zebrafish *mct8* morphants, neurons develop outside of the dorsal spinal cord, a region where the most significant loss of RGCs is observed.

The developmental genetic mechanisms underlying MTH control of development are poorly understood. Notwithstanding, our previous findings indicate that MTH regulates zebrafish neurodevelopment by modulating critical genetic signaling pathways, most notably WNT, SHH, and NOTCH (45). In zebrafish neurodevelopment, the NOTCH pathway appears especially responsive to MTH signaling, as major system components respond time-dependently to the hormone (**Figure 1**, present study). NOTCH plays a fundamental role in regulating animal neurodevelopment (revised in (92)), most notably by lateral inhibition, where it promotes cell fate specification of neural progenitors and daughter cells. However, the only examples of T3 control of the NOTCH pathway come from studies in mice (93) and Xenopus (94) postnatal intestinal development. In these models, T3 regulates several components of the NOTCH pathway, including receptors and ligands, in intestinal progenitor cells in a time and cell-context-dependent manner, hence functioning as a cell fate determinant (93, 94). Our findings point to a similar mode of action of MTH on the NOTCH pathway by regulating neural progenitor proliferation, survival, and developmental output during zebrafish neurodevelopment (discussed above). We use live imaging to show impaired MTH signaling decreases asymmetric divisions during zebrafish spinal cord development while symmetric divisions are unaffected. In the developing nervous system, symmetric divisions are associated with progenitor pool amplification or terminal differentiation of progenitors (55, 56). In contrast, asymmetric divisions are related to the acquisition of new cell fates by daughter cells or asymmetric terminal differentiation giving rise to different daughter cells and, in this way, increasing cell diversity (55). An excellent example is the lack of development of inhibitory *pax8* neurons that are lost in the spinal cord of *mct8* morphants (27). Furthermore, cell-autonomous activation of the NOTCH pathway, accomplished by mosaic overexpression of NICD, cannot rescue the consequences of impaired MTH signaling in neural progenitor cells. This observation reinforces the hypothesis that MTH likely functions in neurodevelopment as an integrative signal that allows for balanced NOTCH signaling that gives rise to the different neural cell types in a time and cell-context-dependent manner and that cannot be rescued in a cell-autonomous manner. The observation that impaired T3 signaling impacts delta and jagged ligands expression in opposing manners ( (45), present study) suggests that the hormone functions as a balance and integrator that enables the appropriate input from NOTCH ligands and the developmental outcome that arises from that. That is of extreme significance given that new studies indicate that NOTCH ligand dynamics are fundamental for mice multipotent pancreatic progenitor cell output and the fate of daughter cells arising from the division of these progenitors (95). Such an integrative function of MT3 in neurodevelopment supports the observations that both excess and impaired hormone signaling have profound effects on central nervous system development and function.

Nonetheless, new studies are necessary to further dissect MTH’s role on NOTCH signaling modulation and neural progenitor output in zebrafish and human neurodevelopment. Another important aspect of this evidence is that MTH action is modulated by tissue and cellular context. Engraftment of human patient-derived *MCT8*(-/-) iPSCs cells into euthyroid neonatal mice corpus callosum and cerebellum can differentiate into oligodendrocytes and myelinate adjacent fibers. In contrast, if these patient-derived cells are injected into an *Mct8*(-/-);*Oatp1c1*(-/-);*Rag2*(-/-) hypothyroid neonatal mice corpus callosum and cerebellum human *MCT8*(-/-) iPSCs cells remain in an undifferentiated progenitor state (96). That is reminiscent of our present results with NOTCH and argues that MTH action in neurodevelopment depends highly on cell and tissue context.

The implications of present findings for the comprehension of ADHS, and the development of putative therapies, are significant. It has been suggested that the pathogenesis associated with MCT8 deficiency arises from impaired TH transport across the blood-brain barrier (97, 98). Here we show that the effect over neural cell progenitors occurs before blood-brain-barrier development in zebrafish, suggesting that MTH entering through Mct8 of CNS-residing cells regulates their development.

In conclusion, our data support that the restricted temporal action of MTH is critical for vertebrate neurodevelopment. MTH acting through Mct8 is essential to sustain neural progenitor cells’ survival and proliferation, allowing them to reach the full potential of cell diversity during neurogenesis and gliogenesis. That is likely achieved by MTH regulation of particular neural progenitors’ developmental output (i.e. fate decisions) reflecting neuronal and glial cell populations. In zebrafish *mct8* morphant embryos, the overall neurodevelopmental effects of MTH impairment arise from the lack of a direct action of MTH on target gene transcription and relief of gene expression repression by unliganded thyroid receptors. In both cases, the likely cause behind this impaired development is decreased differentiated neural cell diversity due to the loss of lineage-committed progenitors. Given this evidence, two non-mutually exclusive hypotheses arise to explain how MTH regulates vertebrate neurodevelopment: 1) MTH acts in neural progenitors to allow particular cellular states that enable the generation of the full potential cell fates arising from these progenitors, and 2) MTH acts by allowing final differentiation and survival of neural progenitors committed to a given cell fate generation.

## Data availability statement

The raw data supporting the conclusions of this article will be made available by the authors, without undue reservation.

## Ethics statement

Fish husbandry was conducted by trained scientists according to the EU Directive (2010/63/EU) and followed the Portuguese legislation for the use of laboratory animals (DL n°113/2013, 7 August). All experimental animals are younger than 5-dpf, and no ethical approval is required accordingly to Portuguese (DL n°113/2013, 7 August) and EU (2010/63/EU) law.

## Author contributions

NS: Methodology, Visualization, Writing - Original Draft, Review and Editing. MC: Conceptualization, Methodology, Writing - Original Draft, Review and Editing, Supervision. All authors contributed to the article and approved the submitted version.

## Supplementary material

Supplementary Figure 1 MCT8 morpholino KD is a valid strategy to generate a zebrafish AHDS biomedical model.

Supplementary Figure 2 Expression of MTH-responsive genes reveals 22-31hpf as the developmental time more sensitive to MTH.

Supplementary Figure 3 Impairment of MTH signaling leads to altered spinal cord cytoarchitecture at the end of embryogenesis.

Supplementary Figure 4 Spatio-temporal expression of MTH cellular signaling genes mct8, thraa, and thrab during zebrafish spinal cord neurodevelopment.

Supplementary Figure 5 Impaired MTH signaling affects mitosis and apoptosis in the spinal cord.

Supplementary Figure 6 Colocalization analysis of *her 2* expression by wish with cell mitosis by immunohistochemistry with phosphohistone 3 (PH3) and apoptotic cell labelling (TUNEL) of a 22hpf CTRLMO zebrafish.

Supplementary Figure 7 Colocalization analysis of *dla* expression by wish with cell mitosis by immunohistochemistry with phosphohistone 3 (PH3) and apoptotic cell labeling (TUNEL) of a 22hpf CTRLMO zebrafish.
