# Corrigendum: In a zebrafish biomedical model of human Allan-Herndon-Dudley syndrome impaired MTH signaling leads to decreased neural cell diversity

**DOI:** 10.3389/fendo.2023.1228183

**Published:** 2023-05-31

**Authors:** Nádia Silva, Marco António Campinho

**Affiliations:** ^1^ Centre for Marine Sciences of the University of the Algarve, Faro, Portugal; ^2^ Algarve Biomedical Center-Research Institute, University of the Algarve, Faro, Portugal; ^3^ Faculty of Medicine and Biomedical Sciences, University of the Algarve, Faro, Portugal

**Keywords:** maternal thyroid hormone, monocarboxylic acid transporter 8, neurodevelopment, spinal cord, zebrafish, Allan-Herndon-Dudley syndrome (AHDS)

In the published article, the Funding statement was missing. The correct Funding statement appears below.

“This study received Portuguese national funds from FCT - Foundation for Science and Technology through project PTDC/EXPL/MAR-BIO/0430/2013 and FCT UIDB/04326/2020 COMPETE 2020, through project EMBRC.PT ALG-01-0145-FEDER-022121. ABC-RI CRESC Algarve 2020. NS was a recipient of an FCT Ph.D. grant SFRH/BD/111226/2015. MC received an FCT-IF Starting Grant (IF/01274/2014). Support of ABC and Camara Municipal de Loulé”.

The authors apologize for this error and state that this does not change the scientific conclusions of the article in any way. The original article has been updated.

