# Supplementary material for: In a zebrafish biomedical model of human Allan-Herndon-Dudley syndrome impaired MTH signaling leads to decreased neural cell diversity
Source: Front Endocrinol (Lausanne). 2023 May 4;14:1157685. doi: 10.3389/fendo.2023.1157685 (PMC10194031; doi:10.3389/fendo.2023.1157685)
Supplement: Supplementary Figure 1 — MCT8 morpholino KD is a valid strategy to generate a zebrafish AHDS biomedical model. [file DataSheet_1.docx]

Supplementary Material

**In a zebrafish biomedical model of human Allan-Herndon-Dudley syndrome impaired MT3 signaling leads to decreased neural cell diversity**

**Nádia Silva and Marco António Campinho***

***Correspondence:** Corresponding Author: macampinho@ualg.pt

# Supplementary Tables

Supplementary Table 1- Specific primers for target genes used for qPCR analysis.

| **Gene** | **Forward primer (5'-3')** | **Reverse primer (5'-3')** | **Product Size (bp)** | **GenBank accession no.** |
| --- | --- | --- | --- | --- |
| ***dla*** | GGCACAGGTAAGAGTTGACCA | CAGTGTCCCACTGGATATGCT | 115 | NM_130954 |
| ***dld*** | GCAGAAATATCCTCCCTCTGG | CCTACAGCTGCACTTGTCCTC | 128 | NM_130955 |
| ***gbx1*** | CAGGTCCAGTTTCTCGTCATC | TCTCGGACCCTCCAGATAGTT | 200 | NM_174861 |
| ***her2*** | ACAGCTGTCAGATCACGTCAG | CGCGCGTGAAGTAAAGCAATA | 197 | XM_005155921 |
| ***her4*** | CCGTCAGCTTTCTGTCTCAGT | TCAGTGGTCTGAGGATTGTCC | 124 | NM_001161409.1 |
| ***hoxb1b*** | CAAGTATCAGGTCTCCCCTGAC | CTTCTCAAGTTCCGTGAGCTG | 190 | NM_131142 |
| ***jag2a*** | GTTGGCTGTGAGAGAGGTCAG | TGCTCTCCTCCATCTGAGTGT | 182 | XM_005156965 |
| ***neurog1*** | CAGATGTAGTTGTGAGCGAAGC | GAGAGGAACAGGATGCACAAC | 125 | AF024535 |
| ***notch1a*** | GTAATGGTGCCACCTGTGTCT | TTGTAGCACGGGTTGACTAGG | 121 | NM_131441 |
| ***notch1b*** | AGGGAAGGAAGTTACGAGACG | AGGACTGCGCACTAGGTTGTA | 159 | NM_131302 |
| ***sox19a*** | ATGTCCCTCAGGTCTCCTTGT | AACTCCTACAACCCCATGTCC | 146 | AB242331.1 |
| ***sox19b*** | GCACTGCTGCTGTAGGACATT | CAACTTCACCGGTACGATCTG | 101 | NM_131702 |
| ***sox3*** | CCTCGTCAATGAAGGGTCTCT | GAAGATGGCTCAGGAGAATCC | 110 | AB117960 |

Supplementary Table 2 - Primers used for cloning of target sequences and use for in situ hybridization riboprobe synthesis, and references of previously published riboprobes.

| **Gene** | **Forward primer (5'-3')** | **Reverse primer (5'-3')** | **Product Size (bp)** | **GenBank accession no.** |
| --- | --- | --- | --- | --- |
| ***neurog1*** | GACTACTCCTTTTCGCACACG | ACGTCGGTTTGCAAGTATCC | 548 | AF024535 |
| ***fabp7a*** | GAACTTCGACGAGTACATGA | CACCATCATTGACATTCTGC | 690 | AY145893 |
| ***slc1a2b*** | GTCACCGTCAGTCTTACAGCA | GGGTTCTTCCTCAACGACTGT | 441 | [NM_199979.2](https://www.ncbi.nlm.nih.gov/nucleotide/NM_199979.2?report=genbank&log$=nucltop&blast_rank=4&RID=0JACM4K0016) |
| ***olig2*** | TGTCCAGCAGACCTTCTTCTC | CACATGCTACACGGACAAGG | 727 | [AF442964.1](https://www.ncbi.nlm.nih.gov/nucleotide/AF442964.1?report=genbank&log$=nucltop&blast_rank=2&RID=0JADPYC4016) |
| ***her2*** | CACACACGCGAGCTCTGACAGC | CACCTCTGCAGGCTACACATCTC | 689 | XM_005155921 |
| ***dla*** | (Haddon et al., 1998) | | | |
| ***thraa*** | (Campinho et al., 2014) | | | |
| ***thrab*** | (Campinho et al., 2014) | | | |
| ***mct8*** | (Campinho et al., 2014) | | | |

## Supplementary Figures


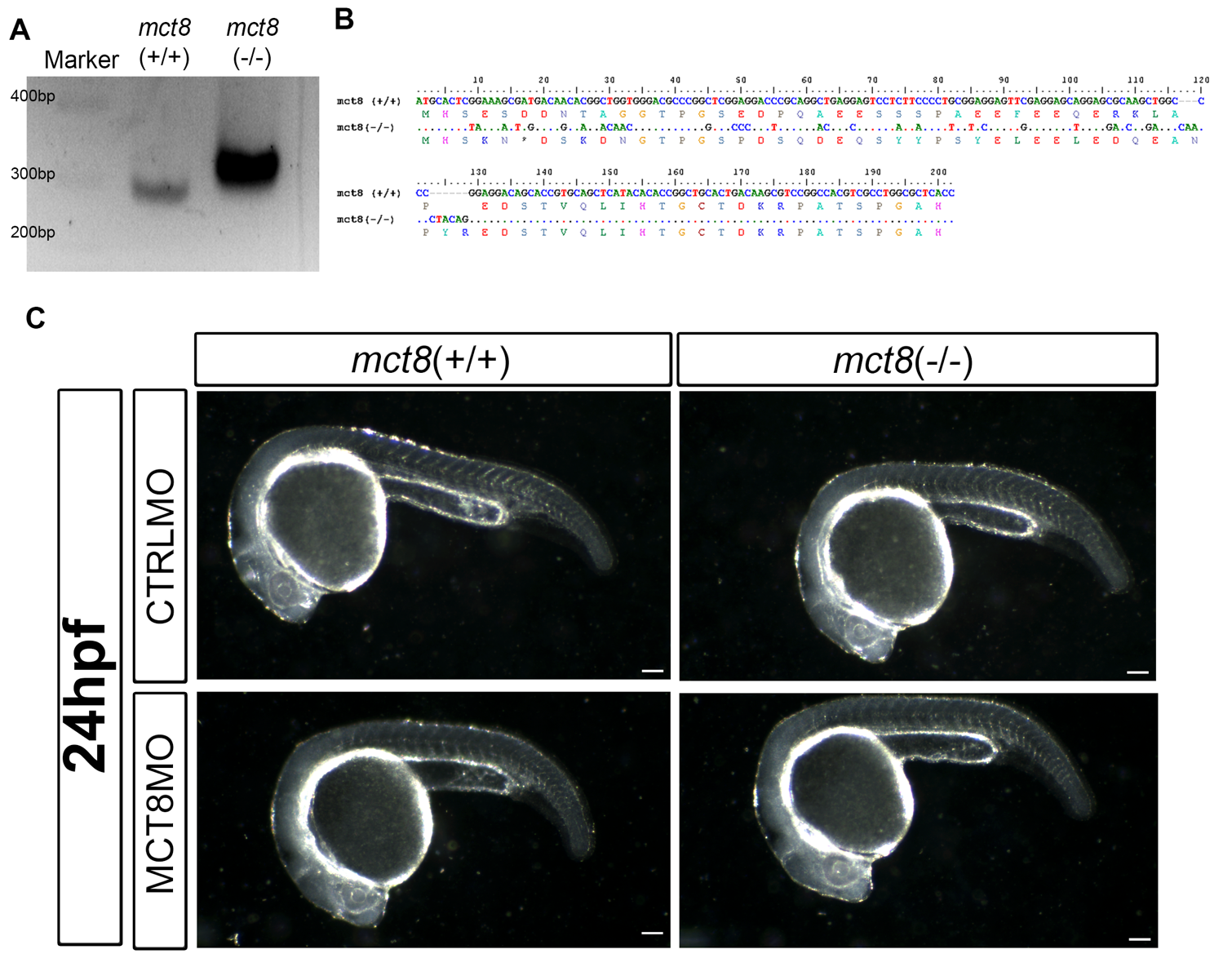


**Supplementary Figure 1 - MCT8 morpholino KD is a valid strategy to generate a zebrafish AHDS biomedical model.** MCT8MO injection in *mct8* loss-of-function mutants (-/-) does not give unspecific effects, further proving that the MCT8 morpholino KD is a valid AHDS biomedical model. **(A)** PCR genotyping of *mct8* (-/-) embryos generate a 9-nt insertion in the first exon of the zebrafish mct8 locus. **(B)** Sanger sequencing of mutant *mct8* band confirms a nonsense mutation in codon 6, several missense mutations, and a 9-nt insertion in the gRNAs binding region of *mct8* exon 1. **(C)** Injection of MCT8MO in *mct8* mutant genetic background does not induce additional effects in 24hpf embryos. *mct8* (-/-) mutant embryos injected with CTRLMO present a phenotype similar to that found in MCT8MO.


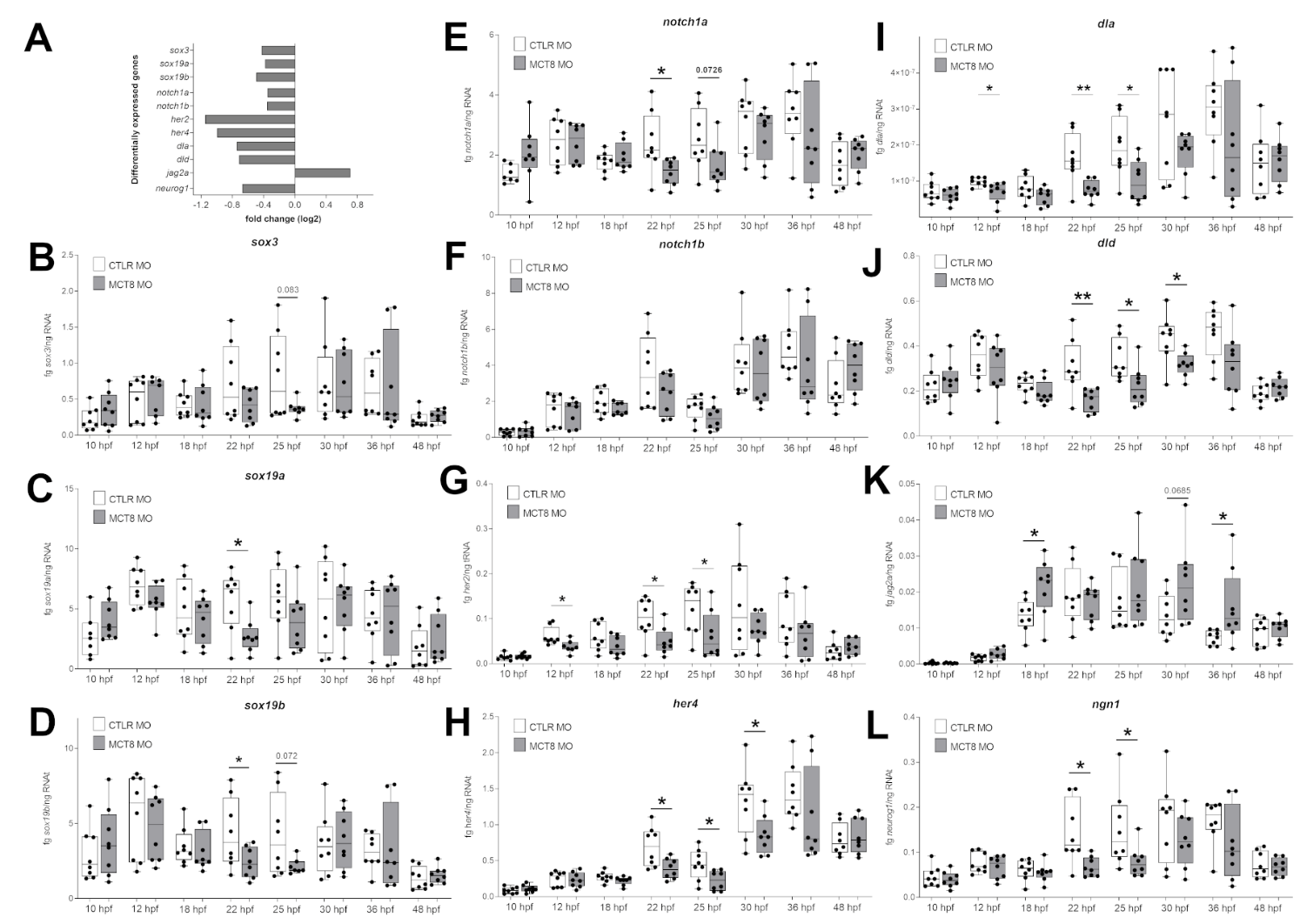


**Supplementary Figure 2 - Expression of MT3-responsive genes reveals 22-31hpf as the developmental time more sensitive to MT3.** **(A)** Quantification of genes of interest, mapped to the neurogenesis cascade, differentially expressed between control and MCT8MO zebrafish, identified by RNA-Seq expressed as Log2 of fold change (n=7, p<0.01, FDR<0.0001) (NCBI–BioProjects: PRJNA381309). **(B-L)** Box-and-whiskers plot of gene expression levels determined after RT-qPCR for *sox3* **(B),** *sox19a* **(C)**, *sox19b* **(D),** *notch1a* **(E)**, *notch1b* **(F)**, *her2* **(G)**, *her4* **(H)**, *dla* **(I)**, *dld* **(J)**, *jag2a* **(K)** and *neurog1* **(L)**. Data is represented as fentograms (fg) of the gene of interest by nanograms (ng) of total RNA used for cDNA preparation. Statistical significance was determined at each time point, comparing gene expression in MCT8MO relative to CTRLMO using a t-test after normal distribution was confirmed (D'Agostino & Pearson test). N = 8 (* p<0.05; ** p<0.01).


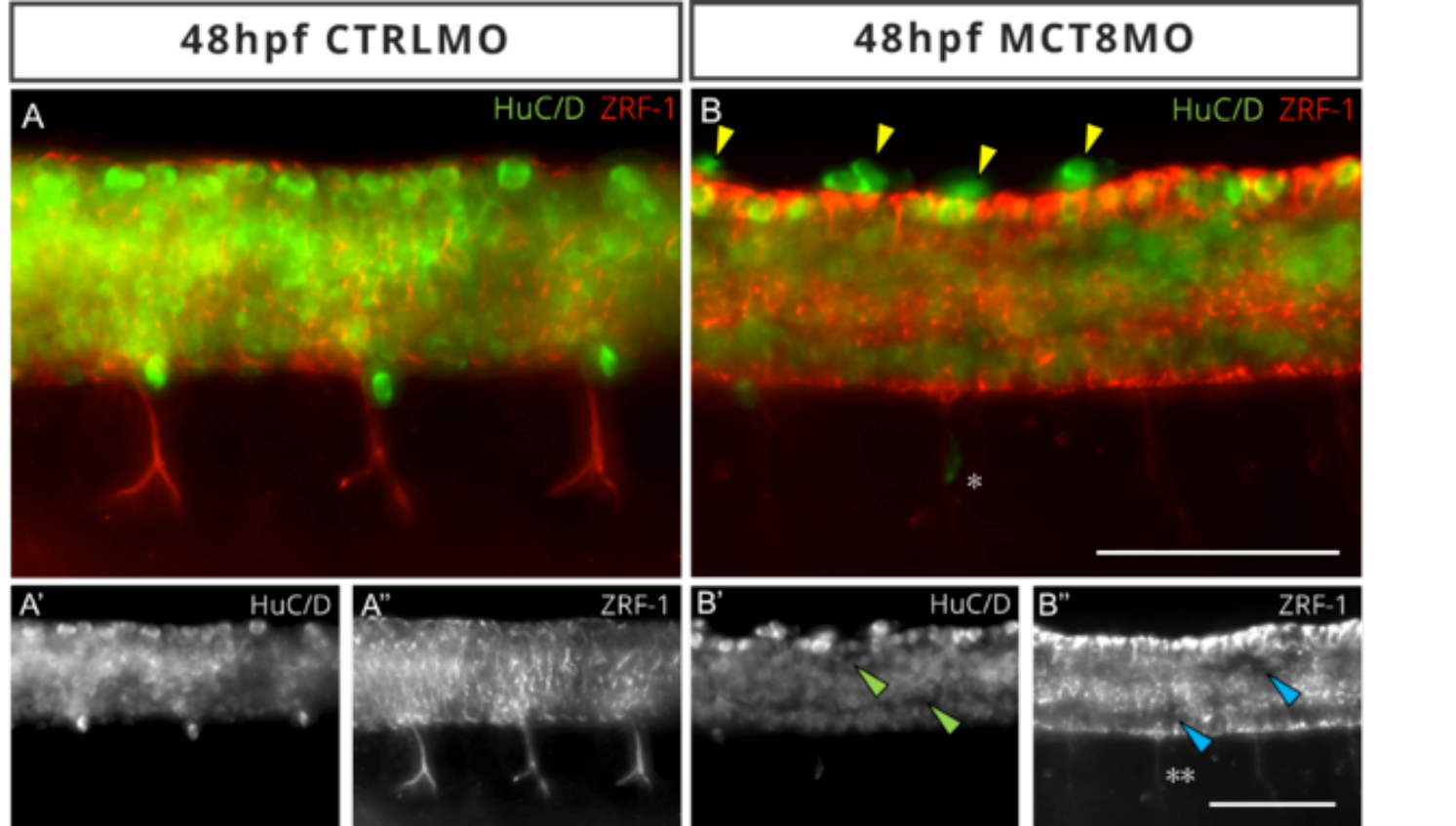


**Supplementary Figure 3 - Impairment of MT3 signaling leads to altered spinal cord cytoarchitecture at the end of embryogenesis.** Characterization of the spinal cord of 48hpf zebrafish by double immunohistochemistry labelling of mature neurons (HUC/D) and glial cells (ZRF-1). **(A)** In CTRLMO embryos, neurons assume a V-shaped organization. **(A')**, and the perineurial glial cells (red) are located adjacent to the motor nerve, descending toward the muscle. Glial cells are well distributed and surround neurons in all directions **(A'')**. **(B)** In MCT8MO embryos, neurons accumulate ectopically outside the dorsal limit of the spinal cord (yellow arrowheads in **B**) and are not distributed equally along the spinal cord (green arrowheads in B'). Perineurial glial cell projections are absent or abnormally extended to the myotome (**). Glial cells are disorganized and have accumulated at the most dorsal and ventral regions leading to "holes" in the spinal cord (blue arrowheads in **B''**). Motoneurons do not migrate normally towards the myotome in MCT8MO embryos (* in **B**). In all images, rostral is left, and dorsal is up. Images are maximum projections of spinal cord z-stacks. The scale bar represents 50 µm.


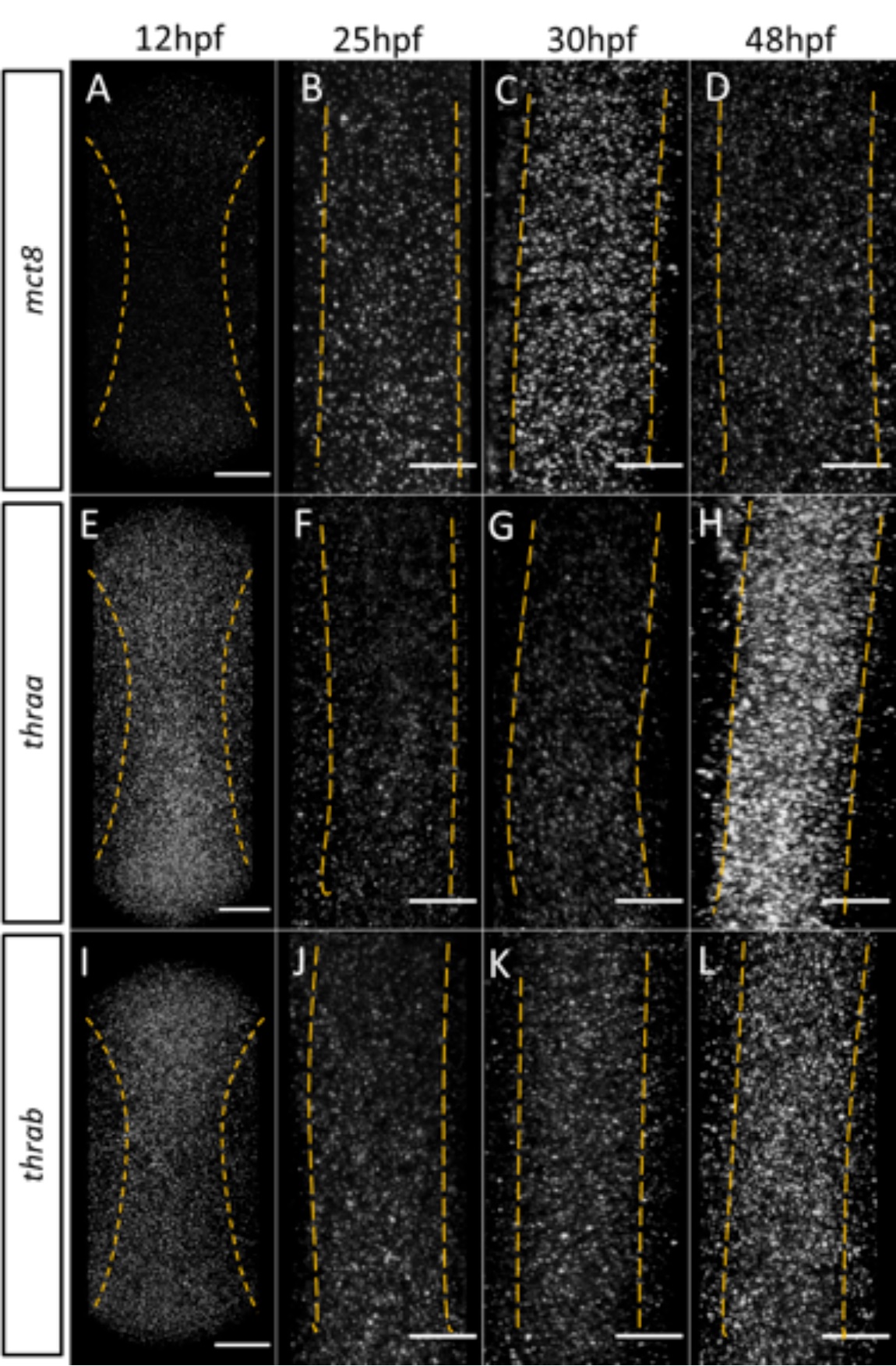


**Supplementary Figure 4 - Spatio-temporal expression of MT3 cellular signaling genes mct8, thraa, and thrab during zebrafish spinal cord neurodevelopment.** Fluorescent WMISH in CTRLMO embryos allowed determination of the spatio-temporal expression pattern of *mct8*, *thraa, and thrab* at 12, 25, 30, and 48hpf*.* Images represent dorsal maximum projections of the whole spinal cord at 12hpf and trunk sections of the spinal cord between somites 8-12 at 25, 30, and 48hpf. Yellow *v*ertical dashed lines show the lateral boundary of the spinal cord. In all images rostral is up. Scale bars represent 25µm, except for 12hpf where the scale bar represents 50 µm.


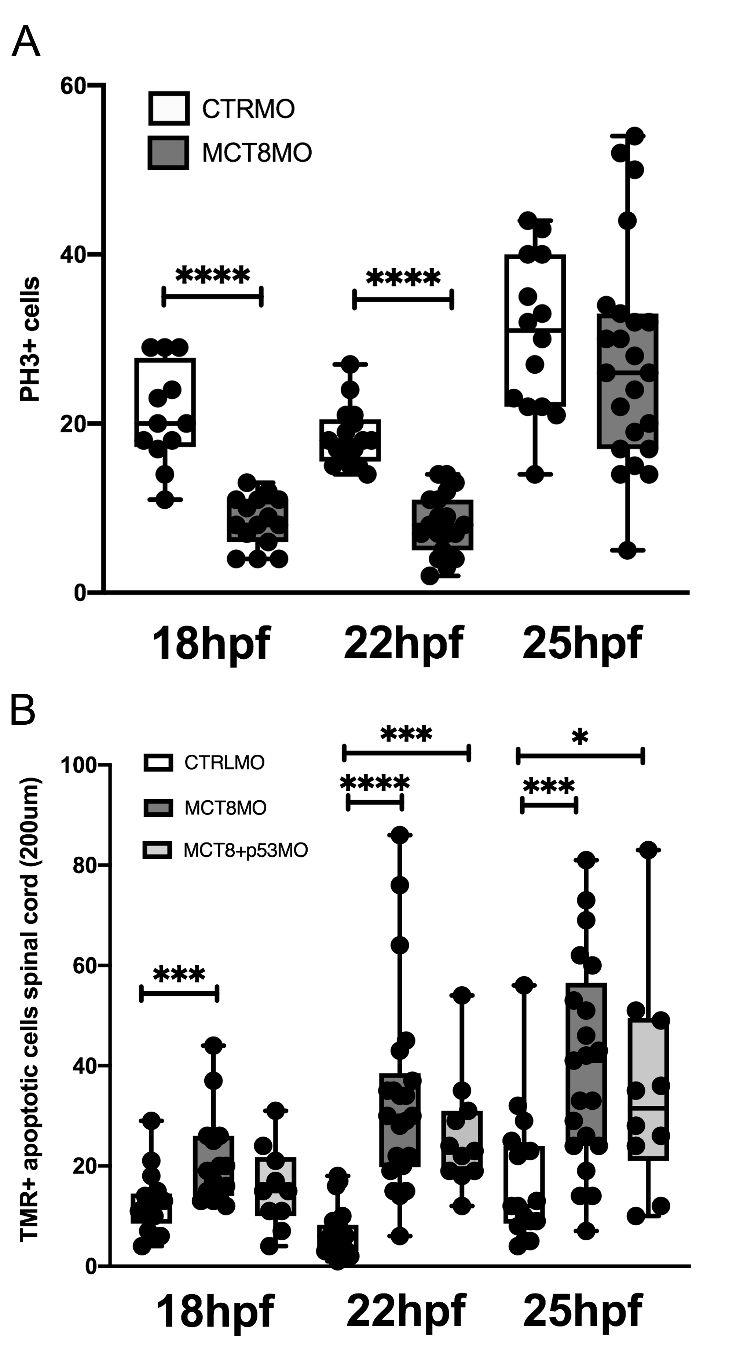


**Supplementary Figure 5 - Impaired MT3 signaling affects mitosis and apoptosis in the spinal cord.** Overall mitotic **(A)** and apoptotic **(B)** cells in the spinal cord of embryos injected with CTRLMO or MCT8MO at 18, 22, and 25hpf. Quantifications were carried out in a 200µm wide spinal cord volume between somite 8-12. In the case of apoptosis, an additional experimental group was co-injected with MCT8MO and p53MO to mitigate the non-specific toxic effects of MCT8MO. No differences were found between MCT8MO and MCT8MO+p53MO groups. Statistical differences were found after unpaired t-test and considered if p≤0.05. * p≤0.05, *** p<0.001, **** p<0.0001.

**
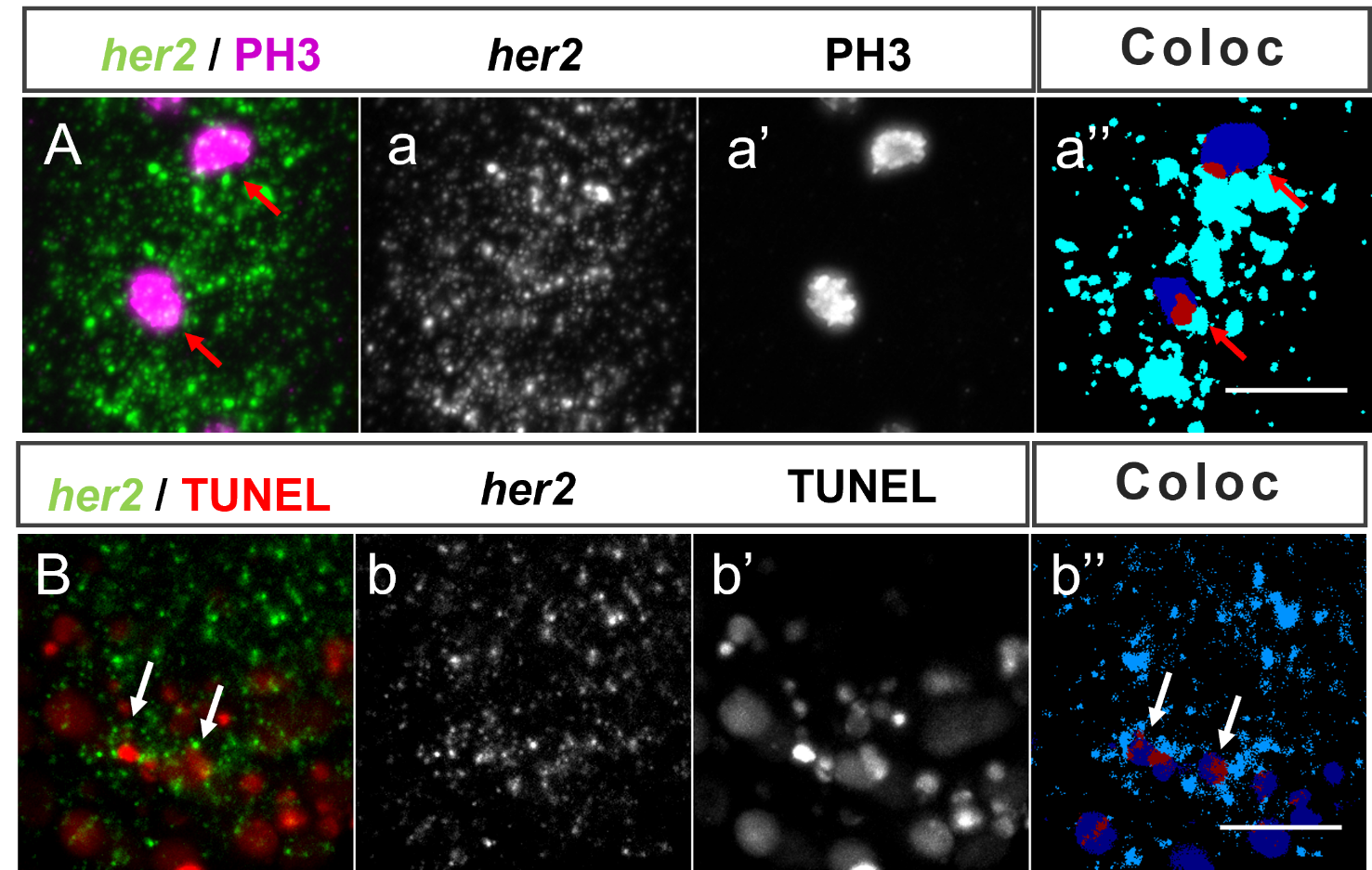
**

**Supplementary Figure 6 -** Colocalization analysis of *her 2* expression by wish with cell mitosis by immunohistochemistry with phosphohistone 3 (PH3) and apoptotic cell labelling (Tunel) of a 22hpf CTRLMO zebrafish. **(A)** Composite maximum intensity projection of co-labelling *her2* and PH3 (white arrows- colocalization); **(a)** *her2*; **(a')** PH3; **(a'')** colocalized areas obtained through the Colocalization colormap plugin (Fiji) are labelled in dark red and red arrows. **(B)** Composite maximum intensity projection of co-labelling *her2* and Tunel assay; **(a)** *her2*; **(a')** Tunel; **(a'')** colocalized areas are labelled in dark red and white arrows. Images were taken from the spinal cord between somite 8-9, using a Z1.lightsheet microscope (Zeiss). Dorsal views and scale bars represent 20 µm.


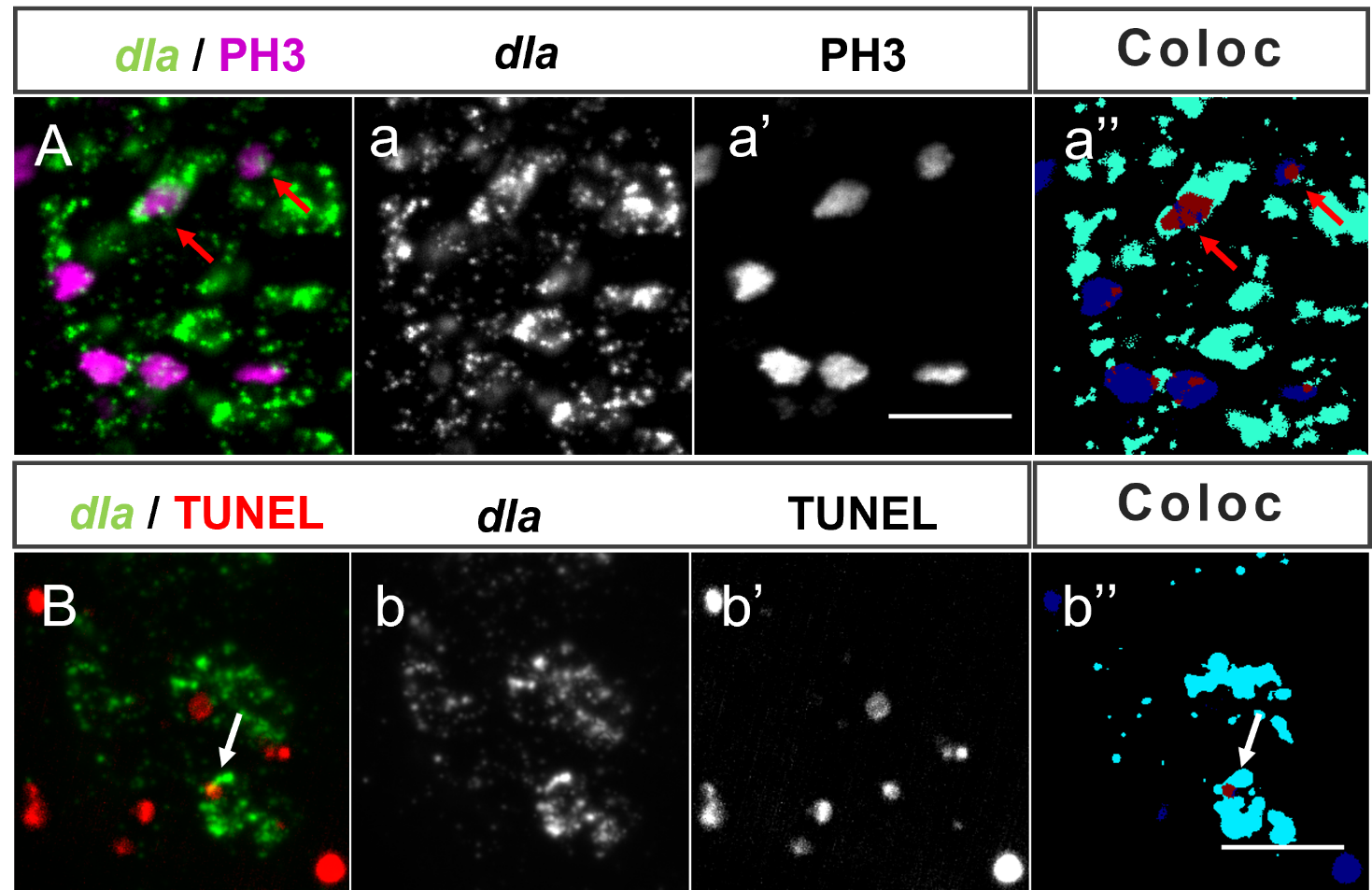


**Supplementary Figure 7 - Impaired MT3 signaling affects mitosis and apoptosis in the spinal cord.** Colocalization analysis of *dla* expression by wish with cell mitosis by immunohistochemistry with phosphohistone 3 (PH3) and apoptotic cell labelling (Tunel) of a 22hpf CTRLMO zebrafish. **(A)** Composite maximum intensity projection of co-labelling *dla* and PH3 (white arrows- colocalization); **(a)** *dla*; **(a')** PH3; **(a'')** colocalised areas obtained through the Colocalization colormap plugin (Fiji) are labelled in dark red and red arrows. **(B)** Composite maximum intensity projection of co-labelling *dla* and Tunel assay; **(a)** *dla*; **(a')** Tunel; **(a'')** colocalized areas are labelled in dark red and white arrows. Images were taken from the spinal cord between somite 8-9, using a Z1.lightsheet microscope (Zeiss). Dorsal views and scale bars represent 20 µm.
